# Interaction Testing and Polygenic Risk Scoring to Estimate the Association of Common Genetic Variants With Treatment Resistance in Schizophrenia

**DOI:** 10.1001/jamapsychiatry.2021.3799

**Published:** 2022-01-12

**Authors:** Antonio F. Pardiñas, Sophie E. Smart, Isabella R. Willcocks, Peter A. Holmans, Charlotte A. Dennison, Amy J. Lynham, Sophie E. Legge, Bernhard T. Baune, Tim B. Bigdeli, Murray J. Cairns, Aiden Corvin, Ayman H. Fanous, Josef Frank, Brian Kelly, Andrew McQuillin, Ingrid Melle, Preben B. Mortensen, Bryan J. Mowry, Carlos N. Pato, Sathish Periyasamy, Marcella Rietschel, Dan Rujescu, Carmen Simonsen, David St Clair, Paul Tooney, Jing Qin Wu, Ole A. Andreassen, Kaarina Kowalec, Patrick F. Sullivan, Robin M. Murray, Michael J. Owen, James H. MacCabe, Michael C. O’Donovan, James T. R. Walters, and the Genetics Workstream of the Schizophrenia Treatment Resistance and Therapeutic Advances (STRATA) Consortium and the Schizophrenia Working Group of the Psychiatric Genomics Consortium (PGC), Olesya Ajnakina, Luis Alameda, Thomas R. E. Barnes, Domenico Berardi, Elena Bonora, Sara Camporesi, Martine Cleusix, Philippe Conus, Benedicto Crespo-Facorro, Giuseppe D’Andrea, Arsime Demjaha, Kim Q. Do, Gillian A. Doody, Chin B. Eap, Aziz Ferchiou, Marta Di Forti, Lorenzo Guidi, Lina Homman, Raoul Jenni, Eileen M. Joyce, Laura Kassoumeri, Inès Khadimallah, Ornella Lastrina, Roberto Muratori, Handan Noyan, Francis A. O’Neill, Baptiste Pignon, Romeo Restellini, Jean-Romain Richard, Franck Schürhoff, Filip Španiel, Andrei Szöke, Ilaria Tarricone, Andrea Tortelli, Alp Üçok, Javier Vázquez-Bourgon

**Affiliations:** 1 MRC Centre for Neuropsychiatric Genetics and Genomics, Division of Psychological Medicine and Clinical Neurosciences, School of Medicine, Cardiff University, Cardiff, United Kingdom; 2 Department of Psychosis Studies, Institute of Psychiatry, Psychology & Neuroscience, King’s College London, London, United Kingdom; 3 Department of Psychiatry, University of Münster, Münster, Germany; 4 Department of Psychiatry, Melbourne Medical School, The University of Melbourne, Melbourne, Australia; 5 The Florey Institute of Neuroscience and Mental Health, The University of Melbourne, Melbourne, Australia; 6 Department of Psychiatry and the Behavioral Sciences, State University of New York Downstate Medical Center, Brooklyn; 7 Institute for Genomic Health, State University of New York Downstate Medical Center, Brooklyn; 8 Department of Psychiatry, Veterans Affairs New York Harbor Healthcare System, Brooklyn; 9 School of Biomedical Sciences and Pharmacy, University of Newcastle, Newcastle, Australia; 10 Centre for Brain and Mental Health Research, University of Newcastle, Newcastle, Australia; 11 Hunter Medical Research Institute, Newcastle, Australia; 12 Neuropsychiatric Genetics Research Group, Department of Psychiatry, Trinity College Dublin, Dublin, Ireland; 13 Department of Genetic Epidemiology in Psychiatry, Central Institute of Mental Health, Medical Faculty Mannheim, University of Heidelberg, Heidelberg, Mannheim, Germany; 14 School of Medicine & Public Health, The University of Newcastle, Newcastle, Australia; 15 Molecular Psychiatry Laboratory, Division of Psychiatry, University College London, London, United Kingdom; 16 Norwegian Centre for Mental Disorders Research, Institute of Clinical Medicine, University of Oslo, Oslo, Norway; 17 Division of Mental Health and Addiction, Institute of Clinical Medicine, Oslo University Hospital, Oslo, Norway; 18 National Centre for Register-based Research, Aarhus University, Aarhus, Denmark; 19 The Lundbeck Foundation Initiative for Integrative Psychiatric Research, Aarhus, Denmark; 20 Queensland Brain Institute, The University of Queensland, Brisbane, Australia; 21 Queensland Centre for Mental Health Research, The University of Queensland, Brisbane, Australia; 22 Department of Psychiatry and Behavioral Sciences, State University of New York Downstate Medical Center, Brooklyn; 23 Department of Psychiatry and Zilkha Neurogenetics Institute, Keck School of Medicine, University of Southern California, Los Angeles; 24 Institute for Genomic Health, State University of New York Downstate Medical Center, Brooklyn; 25 University Clinic and Outpatient Clinic for Psychiatry, Psychotherapy and Psychosomatics, Martin Luther University of Halle-Wittenberg, Halle, Germany; 26 Division of General Psychiatry, Department of Psychiatry and Psychotherapy, Medical University of Vienna, Vienna, Austria; 27 Early Intervention in Psychosis Advisory Unit for South-East Norway, Division of Mental Health and Addiction, Oslo University Hospital, Oslo, Norway; 28 Institute of Medical Sciences, University of Aberdeen, Aberdeen, United Kingdom; 29 Baker Heart and Diabetes Institute, Melbourne, Australia; 30 College of Pharmacy, University of Manitoba, Winnipeg, Manitoba, Canada; 31 Department of Medical Epidemiology and Biostatistics, Karolinska Institutet, Stockholm, Sweden; 32 Department of Psychiatry, Icahn School of Medicine, Mount Sinai Hospital, New York, New York; 33 Department of Genetics, University of North Carolina, Chapel Hill; 34 Department of Biostatistics & Health Informatics, Institute of Psychiatry, Psychology & Neuroscience, King’s College London, University of London, London, United Kingdom; 35 Department of Behavioural Science and Health, Institute of Epidemiology and Health Care, University College London, London, United Kingdom; 36 Department of Psychosis Studies, Institute of Psychiatry, Psychology & Neuroscience, King’s College London, London, United Kingdom; 37 Centro de Investigacion Biomedica en Red de Salud Mental, Spanish Network for Research in Mental Health, Sevilla, Spain; 38 Instituto de Biomedicina de Sevilla, Hospital Universitario Virgen del Rocio, Departamento de Psiquiatria, Universidad de Sevilla, Sevilla, Spain; 39 Treatment and Early Intervention in Psychosis Program, Service of General Psychiatry, Department of Psychiatry, Lausanne University Hospital, Lausanne, Switzerland; 40 Division of Psychiatry, Imperial College London, London, United Kingdom; 41 Department of Biomedical and Neuro-motor Sciences, Psychiatry Unit, Alma Mater Studiorum Università di Bologna, Bologna, Italy; 42 Department of Medical and Surgical Sciences, Bologna Transcultural Psychosomatic Team, Alma Mater Studiorum, University of Bologna, Bologna, Italy; 43 Unit for Research in Schizophrenia, Center for Psychiatric Neuroscience, Department of Psychiatry, Lausanne University Hospital, Lausanne, Switzerland; 44 Department of Medical Education, University of Nottingham Faculty of Medicine and Health Sciences, Nottingham, United Kingdom; 45 Unit of Pharmacogenetics and Clinical Psychopharmacology, Centre for Psychiatric Neuroscience, Department of Psychiatry, Lausanne University Hospital, University of Lausanne, Prilly, Switzerland; 46 School of Pharmaceutical Sciences, University of Geneva, Geneva, Switzerland; 47 Center for Research and Innovation in Clinical Pharmaceutical Sciences, Lausanne University Hospital and University of Lausanne, Lausanne, Switzerland; 48 Institute of Pharmaceutical Sciences of Western Switzerland, University of Geneva, Geneva, Switzerland; 49 University Paris-Est Créteil, Institut national de la santé et de la recherche médicale, Mondor Institute for Biomedical Research, Translational Neuropsychiatry, Fondation FondaMental, Créteil, France; 50 Social Genetics and Developmental Psychiatry, Institute of Psychiatry, Psychology & Neuroscience, King’s College London, London, United Kingdom; 51 South London and Maudsley National Health Service Mental Health Foundation Trust, London, United Kingdom; 52 Department of Social and Welfare Studies, Department of Behavioural Sciences and Learning, Linköping University, Linköping, Sweden; 53 Centre For Public Health, Institute Of Clinical Sciences, Queens University Belfast, Belfast, United Kingdom; 54 UCL Queen Square Institute of Neurology, University College London, London, United Kingdom; 55 Faculty of Social Sciences, Department of Psychology, Beykoz University, Istanbul, Turkey; 56 Assistance Publique–Hôpitaux de Paris, Hôpitaux Universitaires HMondor, Département Médico-Universitaire de Psychiatrie et d’Addictologie, Fédération Hospitalo-Universitaire de Médecine de Précision, Créteil, France; 57 Department of Applied Neuroscience and Neuroimaging, National Institute of Mental Health, Klecany, Czechia; 58 Department of Psychiatry and Medical Psychology, Third Faculty of Medicine, Charles University, Prague, Czechia; 59 Groupe Hospitalier Universitaire Psychiatrie Neurosciences Paris, Pôle Psychiatrie Précarité, Paris, France; 60 Department of Psychiatry, Istanbul University, Istanbul, Turkey; 61 Department of Psychiatry, University Hospital Marques de Valdecilla-Instituto de Investigación Marques de Valdecilla, Santander, Spain; 62 Department of Medicine and Psychiatry, School of Medicine, University of Cantabria, Santander, Spain; 63 Centro de Investigacion Biomedica en Red de Salud Mental, Spanish Network for Research in Mental Health, Santander, Spain

## Abstract

**Importance:**

About 20% to 30% of people with schizophrenia have psychotic symptoms that do not respond adequately to first-line antipsychotic treatment. This clinical presentation, chronic and highly disabling, is known as *treatment-resistant schizophrenia* (TRS). The causes of treatment resistance and their relationships with causes underlying schizophrenia are largely unknown. Adequately powered genetic studies of TRS are scarce because of the difficulty in collecting data from well-characterized TRS cohorts.

**Objective:**

To examine the genetic architecture of TRS through the reassessment of genetic data from schizophrenia studies and its validation in carefully ascertained clinical samples.

**Design, Setting, and Participants:**

Two case-control genome-wide association studies (GWASs) of schizophrenia were performed in which the case samples were defined as individuals with TRS (n = 10 501) and individuals with non-TRS (n = 20 325). The differences in effect sizes for allelic associations were then determined between both studies, the reasoning being such differences reflect treatment resistance instead of schizophrenia. Genotype data were retrieved from the CLOZUK and Psychiatric Genomics Consortium (PGC) schizophrenia studies. The output was validated using polygenic risk score (PRS) profiling of 2 independent schizophrenia cohorts with TRS and non-TRS: a prevalence sample with 817 individuals (Cardiff Cognition in Schizophrenia [CardiffCOGS]) and an incidence sample with 563 individuals (Genetics Workstream of the Schizophrenia Treatment Resistance and Therapeutic Advances [STRATA-G]).

**Main Outcomes and Measures:**

GWAS of treatment resistance in schizophrenia. The results of the GWAS were compared with complex polygenic traits through a genetic correlation approach and were used for PRS analysis on the independent validation cohorts using the same TRS definition.

**Results:**

The study included a total of 85 490 participants (48 635 [56.9%] male) in its GWAS stage and 1380 participants (859 [62.2%] male) in its PRS validation stage. Treatment resistance in schizophrenia emerged as a polygenic trait with detectable heritability (1% to 4%), and several traits related to intelligence and cognition were found to be genetically correlated with it (genetic correlation, 0.41-0.69). PRS analysis in the CardiffCOGS prevalence sample showed a positive association between TRS and a history of taking clozapine (r^2^ = 2.03%; *P* = .001), which was replicated in the STRATA-G incidence sample (r^2^ = 1.09%; *P* = .04).

**Conclusions and Relevance:**

In this GWAS, common genetic variants were differentially associated with TRS, and these associations may have been obscured through the amalgamation of large GWAS samples in previous studies of broadly defined schizophrenia. Findings of this study suggest the validity of meta-analytic approaches for studies on patient outcomes, including treatment resistance.

## Key Points

**Question:**

Can common genetic variants be used to differentiate between treatment-resistant schizophrenia (TRS) and other forms of this disorder?

**Findings:**

Data from this genome-wide association study including 85 490 participants were used to estimate genome-wide single-nucleotide variation effect size differences between individuals with and without TRS, which were compatible with a polygenic model of treatment resistance. Results were used to generate a polygenic risk score, which was significantly associated with TRS status in independent incidence and prevalence samples.

**Meaning:**

Findings of this study based on common genetic variants indicate that TRS is heritable with a modest but significant single-nucleotide variation–based heritability.

## Teaser

This genome-wide association study examines the genetic architecture of treatment-resistant schizophrenia using genetic data from schizophrenia studies and generates a polygenic risk score for TRS status.

## Introduction

Precision psychiatry provides a potential pathway to improve psychiatric classification and develop treatments that are better tailored to specific patients. Major advances in determining the role of genetic variation in the risk of developing psychiatric disorders are helping realize this potential,^1^ but the relevance of these findings to patient outcomes and response to treatment is unclear. Evidence from nonpsychiatric disorders points to distinct genetic bases for disorder susceptibility and prognosis,^2,3^ although few sufficiently powered psychiatric genetic studies have been conducted investigating disorder progression or outcome.^4^ Such study designs could offer valuable insights as to the feasibility of precision psychiatry approaches and their potential impact for patients who have poor outcomes because of the lack of effectiveness of current treatments.^5^

Some of the most disadvantaged individuals in this respect are those with a diagnosis of schizophrenia whose symptoms do not respond adequately to conventional antipsychotic medication.^6,7,8^ This clinical picture is known as treatment-resistant schizophrenia (TRS), which affects approximately 20% to 30% of people with this disorder. The only licensed treatment for TRS is clozapine, which is effective in approximately 60% of cases^9^ and improves most indicators of morbidity and mortality.^10,11^ While the mechanism of action of clozapine is still not fully understood,^12^ it has been proposed that its efficacy might be related to the biological underpinnings of treatment resistance, suggesting a distinct neurobiological etiology to that of non-TRS.^13^ Additionally, several studies have shown that delay in clozapine prescription is associated with resistance even to clozapine.^14,15^ This makes early identification of TRS critical and the ascertainment of correlates of TRS a priority for the field of schizophrenia research.

To date, there is considerable heterogeneity in the findings of genetic studies related to TRS. A family history of schizophrenia is likely associated with developing TRS,^16^ but a recent systematic review did not find any individual genes robustly and specifically associated with this condition.^17^ Researchers have also investigated the long-standing hypothesis that TRS is a more severe form of schizophrenia resulting from a large burden of schizophrenia risk alleles.^18^ While 2 studies reported that aggregating risk variants into a polygenic risk score (PRS) revealed small differences between TRS and non-TRS samples,^19,20^ other analyses did not replicate this result.^16,21,22,23^ However, because each study used slightly different recruitment criteria and definitions of TRS, heterogeneity in the results is expected,^24^ making their collective interpretation difficult. This limitation likely reflects the previously discussed problem of individuals with treatment-resistant psychiatric symptoms being disproportionately underrepresented in research studies owing to poor health, limited capacity to consent, and other causes of attrition, including therapeutic pessimism,^25^ which have precluded the execution of well-powered genome-wide association studies (GWASs).^26^

In this study, we aim to characterize the contribution of common genetic variants to treatment resistance in schizophrenia by exploiting data generated by large consortium-based GWASs of schizophrenia,^27,28^ in which individuals with TRS can be formally defined. Our main hypotheses are that schizophrenia risk alleles will show different genetic associations in the analysis of individuals with and without TRS and that these differences reflect the underlying genetics of treatment resistance. When assessed at a genome-wide level, such differences could reveal commonalities with other complex traits or be validated in clinical cohorts using the PRS approach,^29^ helping us to better understand the biological and epidemiological characteristics of treatment resistance in schizophrenia.^17^

## Methods

For our main analysis, we used samples from large genomic studies of schizophrenia.^27,28^ Some of the largest cohorts in these research initiatives were recruited based on clozapine prescription (a proxy of TRS status), and forming a case-case data set from them would require avoiding confounding factors, such as GWAS batch effects^30^ or population stratification,^31^ which are difficult to control in a multiple-cohort design.^32^ As a safeguard against these, we have used a meta-analytic procedure to assess the differences between GWAS in which individuals with TRS and non-TRS have been compared with matched sets of healthy controls, before comparing the allelic association effect sizes of these 2 GWASs on a genome-wide basis to create a GWAS specific to treatment resistance.

### Genetic Samples and Analysis

We used the CLOZUK1 and CLOZUK2 cohorts as our primary source of individuals with treatment-resistant symptoms, with a total sample size of 10 501 individuals with TRS and 24 542 controls. These cohorts have been described in previous studies.^28,33^ All the individuals with TRS in these samples were prescribed clozapine in the UK after failure of at least 2 trials of antipsychotics, following National Institute for Health and Care Excellence guidelines for TRS.^34^ The use of a history of taking clozapine as equivalent to a research diagnosis of TRS has been validated in these samples,^28^ as well as in independent studies.^35,36^ Control individuals were collected from public databases or through collaboration with population sequencing projects in the UK.

To identify individuals with non-TRS, we used 34 studies included in the meta-analysis by the Schizophrenia Working Group of the Psychiatric Genomics Consortium (PGC),^27^ with a total sample size of 20 325 individuals with schizophrenia and 30 122 controls. In 14 of these studies, available clinical records allowed us to identify and remove all individuals with TRS (eMethods in Supplement 1). The remaining 20 studies were not screened as comparable data were not available, and thus, we conservatively included these samples in the analysis as non–treatment-resistant cases (eTable 1 in Supplement 1). Healthy control individuals in this analysis were a mixture of publicly available samples and clinically ascertained (nonpsychiatric) individuals, and extensive analyses to discard population outliers and assess stratification were also carried out.^27^

GWASs of individuals with TRS vs controls and individuals with non-TRS vs controls were carried out separately using the logistic regression model implemented in PLINK version 2.^37^ Further details on the imputation, quality control, and association testing procedures are described in the eMethods in Supplement 1.

All studies used in the GWAS were reviewed and approved by their local ethical committee. Written informed consent (or legal guardian consent and participant assent) was obtained for all study participants except for those in the CLOZUK cohort, as this study used anonymized blood samples as approved by the UK Multicenter Research Ethics Committee.

### Statistical Analysis

#### Comparing the TRS and Non-TRS Association Studies

To generate association statistics that reflect TRS vs non-TRS differences, we used the test for interaction proposed by Altman and Bland,^38^ which is analogous to a fixed-effect test for moderators in the meta-analytic setting.^39^ This test allows us to calculate an estimate of the difference between 2 odds ratios (ORs), which in our case were those of individuals with TRS vs controls (OR_1_) and those with non-TRS vs controls (OR_2_). The difference, in the scale of the regression β coefficient, equates to difference = log(OR_1_) − log(OR_2_), with standard error SE(d) = √(SE[OR_1_]^2^ + SE[OR_2_]^2^). We transformed this effect size into a *z* score and calculated its associated *P* value at each overlapping single-nucleotide variation (SNV; formerly single-nucleotide polymorphism) between the TRS and non-TRS GWAS, excluding 146 SNVs with a minor allele frequency difference greater than 20% between both data sets to mirror preimputation quality control. For consistency, we also used the same definition of OR_1_ and OR_2_ in all our tests to preserve directions of effect, and thus, positive *z* scores in the interaction analysis reflect stronger SNV associations in individuals with TRS compared with non-TRS.

#### Estimating Heritability

SNV-based heritability was estimated from the TRS interaction summary statistics using LD SCore version 1.01,^40^ after restricting to markers present in the HapMap3 study^41^ and a precomputed linkage disequilibrium reference panel based on the 1000 Genomes phase 3 samples. As point estimates of SNV-based heritability can differ based on model assumptions, we also estimated this quantity via the SumHer framework implemented in LDAK5,^42^ using a default parameter to define the relationship between minor allele frequency and effect size (α = −.25) and multiplicative inflation correction (genomic control). The LD reference for LDAK was estimated from the European samples of the public Haplotype Reference Consortium panel.^43^

#### Polygenic Validation

We sought to investigate whether a TRS GWAS PRS was associated with treatment resistance status in 2 independent samples: one from a schizophrenia prevalence cohort (Cardiff Cognition in Schizophrenia [CardiffCOGS]) and the other from a first-episode incidence cohort (Genetics Workstream of the Schizophrenia Treatment Resistance and Therapeutic Advances [STRATA-G]). The CardiffCOGS prevalence cohort is a sample of individuals with schizophrenia that included both individuals with TRS and treatment-responsive schizophrenia, defined here again from a history of taking or not taking clozapine (n = 817; 315 with TRS and 502 with non-TRS). This is a cross-sectional study with detailed clinical ratings based on research diagnostic interviews and contemporaneous records, as previously described.^44^ We also used a new multiancestry incidence cohort of people with first-episode psychosis, the STRATA-G consortium, in which the participants have been followed up for at least 1 year after initial presentation to ascertain diagnosis and treatment response (eMethods in Supplement 1). As both the first-episode and broad psychosis nature of this sample likely make it diagnostically heterogeneous, we first restricted our analyses to individuals with a diagnosis of schizophrenia at the last time of follow-up. TRS was rated as the presence or absence of a history of taking clozapine (n = 562; 71 with TRS and 492 with non-TRS) to best mirror our TRS GWAS and the definition used in the prevalence cohort. Further details about the recruitment and phenotyping of the STRATA-G sample as well as details of the genotyping and imputation procedure for both data sets are given in the eMethods and eTable 2 in Supplement 1.

The CardiffCOGS study had the relevant UK National Health Service ethical approval, and studies included in STRATA-G were reviewed and approved by their local ethical committees. Written informed consent was obtained for all participants.

#### PRS Analysis

Our primary PRS analysis used the TRS interaction GWAS summary statistics as training set, and the prevalence and incidence cohorts as testing sets where their treatment-resistance phenotype was tested for association. We also investigated the association of PRS and treatment resistance status based on (1) the CLOZUK TRS vs healthy control GWAS and (2) the PGC non-TRS GWAS. Polygenic scores were estimated using PRSice-2 version 2.35^45^ and PRS-CS version June 4, 2021,^46^ as detailed in the eMethods in Supplement 1. Within each pairing of training and testing set, PRS association *P* values were corrected for multiple testing using the Benjamini and Hochberg false discovery rate (FDR) method^47^ as implemented in the R framework *stats* package.

#### Estimating Genetic Correlation and Colocalization

Correlations between treatment resistance and other complex traits were computed using LD-Hub version 1.93,^48^ again based on the LD-Score regression^40^ framework. We only performed the analysis for GWAS categories that had significant phenome-wide associations with psychiatric disorders, based on previous research.^49^ The categories chosen were education, cognitive, personality, psychiatric, and smoking, for a total of 28 summary statistics. Multiple testing correction of these results was also carried out using FDR, since most of the summary statistics tested have overlapping samples and thus yield partially dependent *P* values.

For all associations, statistical significance was set at *P* < .05. All *P *values were 2-tailed.

## Results

### TRS/Non-TRS GWAS

**Figure 1.**
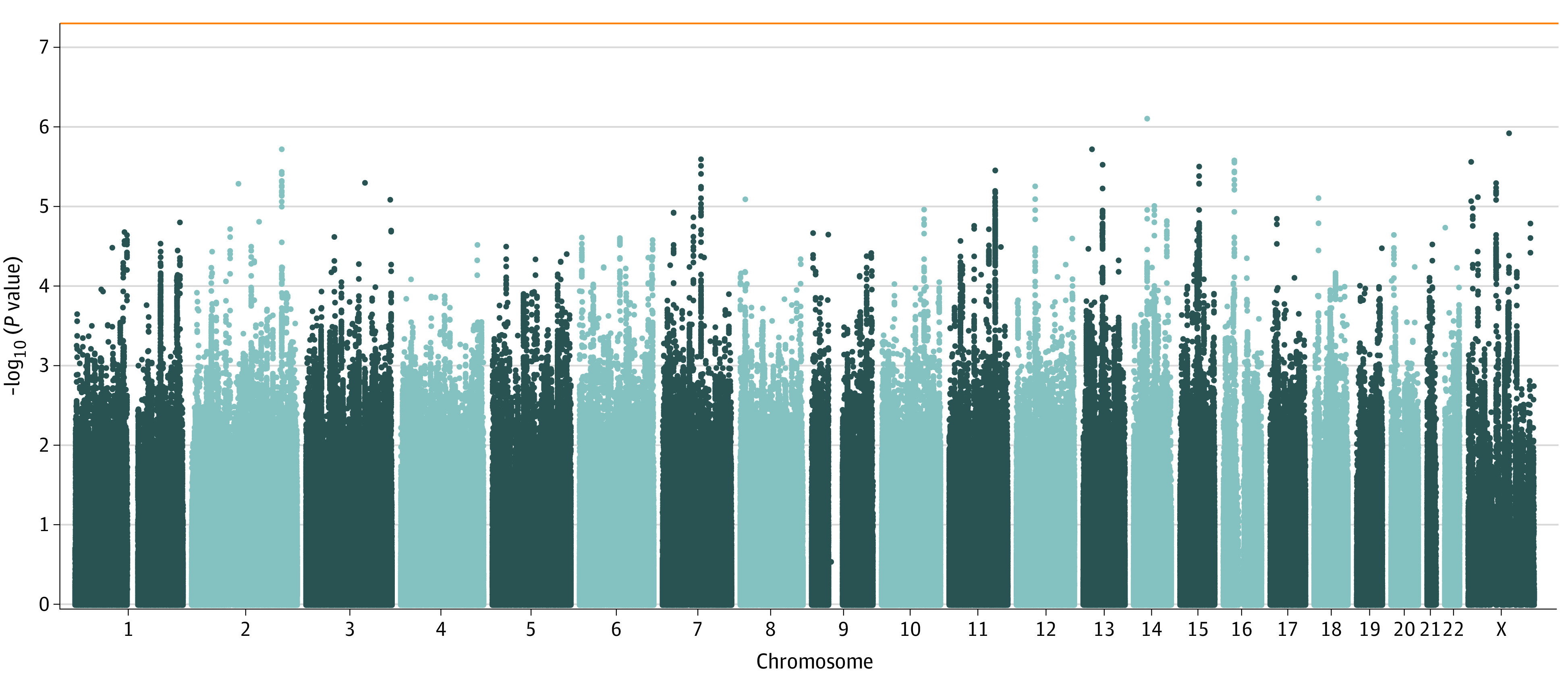
Manhattan Plot of the Treatment-Resistant Schizophrenia (TRS) vs Non-TRS Interaction Genome-Wide Association Study

The study included a total of 85 490 participants (48 635 [56.9%] male) in its GWAS stage and 1380 participants (859 [62.2%] male) in its PRS validation stage. Discovery GWAS of individuals with TRS and non-TRS against independent controls showed highly consistent genome-wide significant signals (eFigure 1 in Supplement 1), all reported in previous schizophrenia studies. This consistency extended to most of the polygenic architecture, as shown by a high genetic correlation estimate between the data sets (r = 0.966; SE, 0.037; *P* = 1.43 × 10^−147^) in which the 95% CI includes 1. No genome-wide SNVs were detected by the TRS interaction analysis (Figure 1), although a slight departure from normality was detected in the genome-wide Q-Q plot (λ = 1.062; λ_1000_ = 1.002; LD-Score intercept, 1.032; SE, 0.007; LDAK scaling estimate, 1.017; SE, 0.010; eFigure 2 in Supplement 1). This is compatible with a polygenic signal,^50^ as the 2 discovery GWASs did not show evidence of inflation caused by other confounding factors.^27,28^ Indeed, the 2 methods we used to estimate the common variant contribution to the observed-scale heritability of TRS returned small values of similar magnitude (SNV-based heritability: LD-Score, 0.013; SE, 0.006; SNV-based heritability: LDAK, 0.040; SE, 0.014).

### Polygenic Score–Based Prediction of TRS in Independent Samples

**Figure 2.**
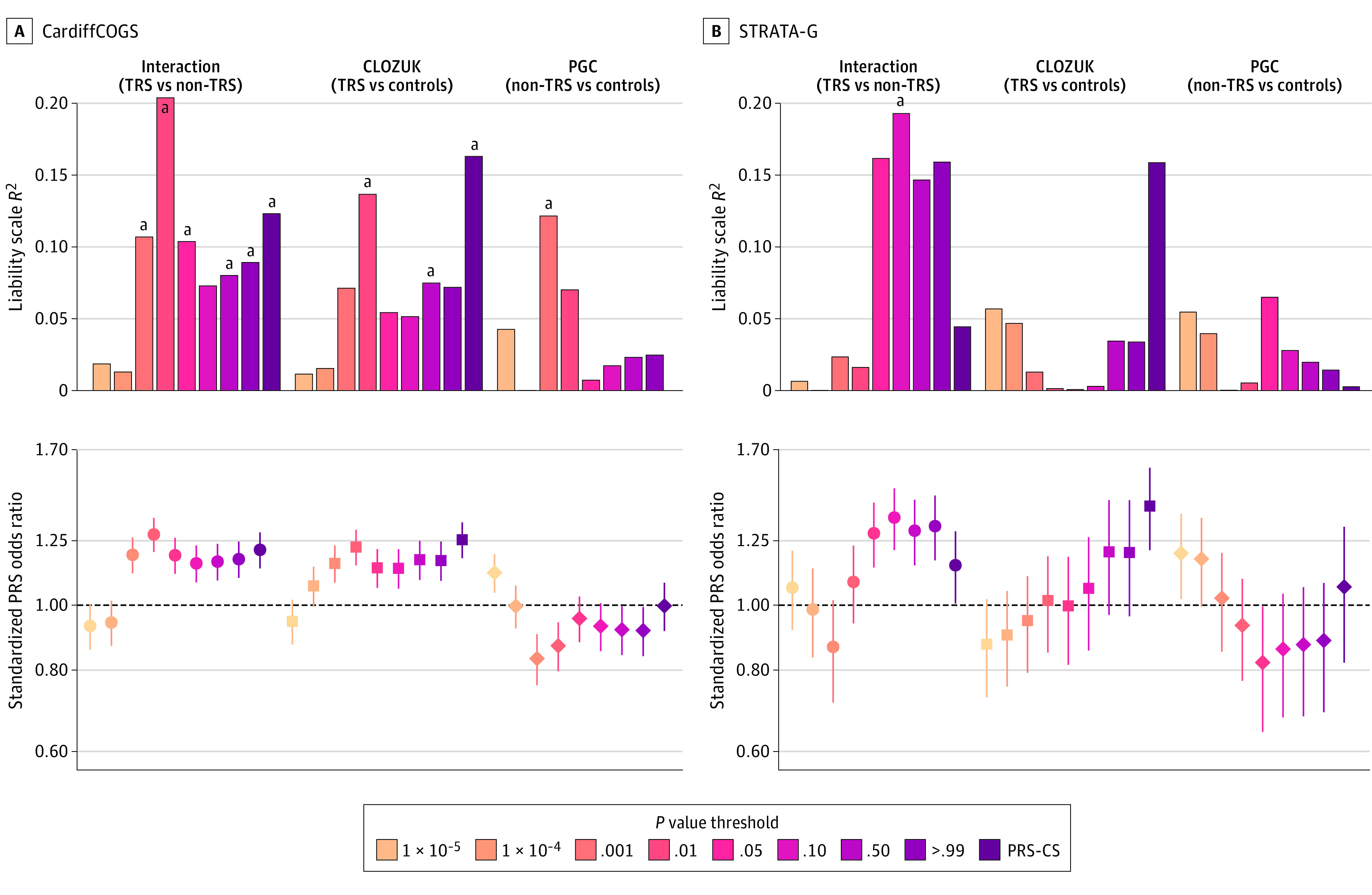
Polygenic Score Analysis of the Cardiff Cognition in Schizophrenia (CardiffCOGS) Cohort and Genetics Workstream of the Schizophrenia Treatment Resistance and Therapeutic Advances (STRATA-G) Cohort Using 3 Different Schizophrenia-Related Polygenic Risk Scores (PRS) Associations between PRS and treatment resistance in schizophrenia (TRS) were defined as a history of taking clozapine in people with a schizophrenia diagnosis. There were 315 individuals with TRS and 502 individuals with non-TRS in the CardiffCOGS cohort and 71 individuals with TRS and 492 control individuals in the STRATA-G cohort. PGC indicates Psychiatric Genomics Consortium; PRS-CS, polygenic prediction via Bayesian regression and continuous shrinkage priors. Whiskers indicate 95% CIs. ^a^*P* < .05.

A PRS generated from the TRS interaction GWAS was positively associated with treatment resistance in our validation prevalence cohort (CardiffCOGS), explaining up to 2.03% of the variance on the liability scale of treatment resistance (*P* = .001; FDR *P* = .01; Figure 2; eTable 3 in Supplement 1). Analyzing the 2 GWASs used in the interaction analysis, we found that a PRS based on CLOZUK GWAS results (TRS vs healthy controls) also explained a significant proportion of the variance in the TRS phenotype (maximum r^2^ = 1.63%; *P* = .004; FDR *P* = .03), very similar to that obtained by testing the larger PGC non-TRS sample (maximum r^2^ = 1.21%; *P* = .01; FDR *P* = .10). However, the effect sizes of these PRSs were generally in opposite directions (Figure 2; eTable 3 in Supplement 1); ie, those taking clozapine in CardiffCOGS had higher PRS derived from the TRS GWAS (OR, 1.22; 95% CI, 1.05-1.41) but a lower PRS from the non-TRS GWAS (OR, 0.83; 95% CI, 0.72-0.96). Focusing on the polygenic prediction via Bayesian regression and continuous shrinkage priors estimates, which do not require LD clumping or *P* value thresholding,^51^ we also observed a significant and positive difference between the effect sizes of the TRS interaction and CLOZUK PRS, while the PGC PRS had a null effect (Figure 2). This pattern of results in an independent case-case comparison is consistent with our interaction analysis detecting a true polygenic signal for treatment resistance, with a greater burden of risk alleles from both this and the CLOZUK TRS GWAS associating with a history of clozapine prescription. Alternatively, the PRS derived from non-TRS samples is not as clearly associated with treatment resistance, and some results of these analyses are even compatible with its enrichment in individuals with non-TRS.

Generation of PRS and their analysis within the first-episode incidence sample (STRATA-G) showed that a greater interaction PRS burden was also associated with having treatment resistance in this setting, explaining up to 1.09% of the variance (*P* = .04; FDR *P* = .21). In contrast, neither the CLOZUK nor PGC PRS were significantly associated with treatment resistance across any of the tested *P* value thresholds or using polygenic prediction via Bayesian regression and continuous shrinkage priors (Figure 2; eTable 3 in Supplement 1). However, a meta-analysis of both CardiffCOGS and STRATA-G resulted in narrower confidence intervals around all the PRS effect size estimates previously estimated from CardiffCOGS, supporting the overall consistency in directions of association between these samples (eFigure 3 and eTable 3 in Supplement 1).

**Figure 3.**
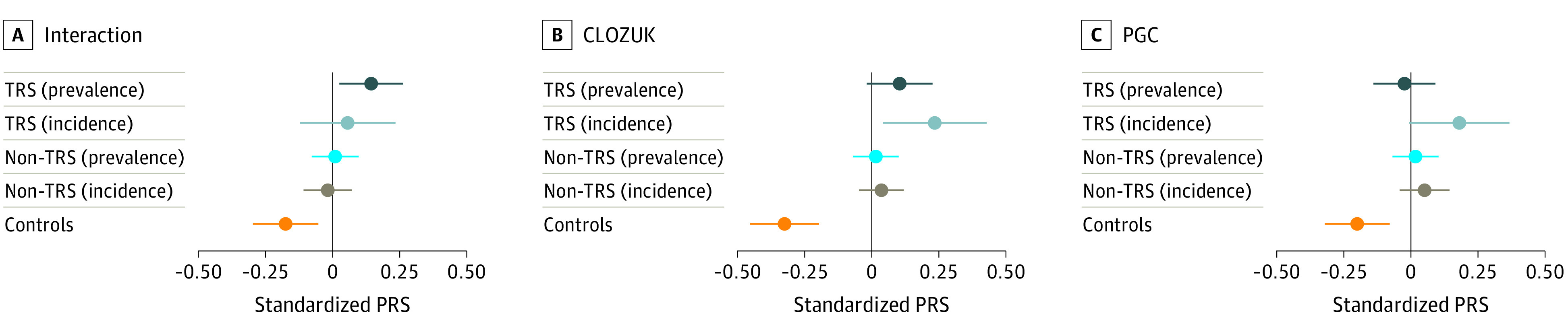
Polygenic Profile Derived From Combined Genotype Data From the Cardiff Cognition in Schizophrenia (CardiffCOGS) and Genetics Workstream of the Schizophrenia Treatment Resistance and Therapeutic Advances (STRATA-G) Cohorts Using Scores Calculated With Linkage Disequilibrium–Independent (*P* < 1) Single-Nucleotide Variants Dots indicate the mean values of the standardized polygenic risk score (PRS; corrected for sex, relevant principal components, and ancestry-informative marker–based ancestry estimates). Whiskers delimit the 95% CIs of the PRS means. PGC indicates Psychiatric Genomics Consortium; TRS, treatment-resistant schizophrenia.

To interpret the results from the prevalence and incidence cohorts alongside each other, we merged the genotyped SNVs from both samples (to a total of 199 074 SNVs) and estimated polygenic scores using all LD-independent SNVs (*P* < 1). As a reference point for this analysis, we retained a small subset of 242 screened unaffected control individuals that had been genotyped as part of the Genetics and Psychosis project,^26^ one of the STRATA-G cohorts. Splitting the combined sample by TRS status showed that the polygenic profile of treatment resistance is largely consistent between CardiffCOGS and STRATA-G, with the TRS interaction PRS burden showing no difference between incident and prevalent TRS individuals (Figure 3). However, we noted a distinction in terms of general schizophrenia risk alleles, as indexed by the CLOZUK and PGC PRS, which were enriched within the individuals with first-episode TRS compared with the individuals with TRS from our cross-sectional sample (CLOZUK: OR, 1.52; SE, 0.21; *P* = .047; PGC: OR, 3.48; SE, 0.39; *P* = 7.69 × 10^−4^). None of the scores we tested were significantly different between the 2 groups of individuals with non-TRS, and all individuals with schizophrenia showed larger mean scores than the unaffected controls.

### Genetic Correlation Between TRS and Other Traits

**Figure 4.**
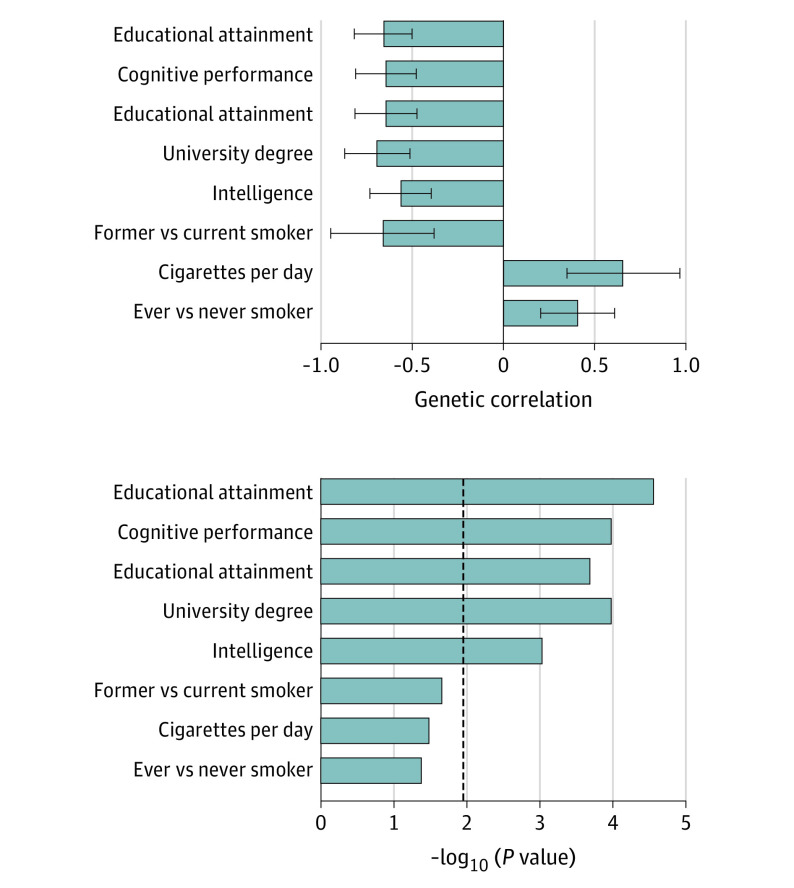
Linkage Disequilibrium Score Regression Genetic Correlation Results of LD-HUB Phenotypes With the Treatment-Resistant Schizophrenia Interaction Genome-Wide Association Study Top, Genetic correlation coefficients shown with standard errors. Bottom, Genetic correlation *P* values. The dashed line indicates a false discovery rate–corrected significant level of *P* = .05.

We used the results of the TRS interaction GWAS to examine the genetic associations between the treatment-resistant phenotype and other disorders and traits. In these analyses, 8 of 28 publicly available GWAS summary statistics displayed nominally significant (*P* < .05) genetic correlations with TRS, of which 5 survived multiple testing correction (FDER p < 0.05; Figure 4; eTable 4 in Supplement 1). All 5 genetically correlated phenotypes were associated with cognitive measures and educational attainment (genetic r, 0.41-0.69), showing our GWAS of TRS to be genetically correlated with lower cognitive ability and lower educational attainment.

## Discussion

We report a large study of treatment resistance in schizophrenia, showing that common genetic variants were associated with this presentation, that these variants were also associated with general risk of schizophrenia, and that the difference in their associations with the 2 phenotypes may have influenced the polygenic score analyses. Furthermore, our results, particularly the large genetic correlation between the TRS and non-TRS GWASs, are consistent with the risk conferred by common SNVs being similar regardless of whether individuals respond to first-line treatments. This suggests that treatment resistance, at least to the extent that we have been able to define it and explore it within this study, is not likely to index a cluster of individuals with a range of etiologies and pathophysiologies fundamentally different from those that contribute to schizophrenia more widely. However, by also assessing differences at the level of individual allelic associations, our results also support the existence of a polygenic contribution associated with treatment resistance that seems largely distinct from liability to schizophrenia. While this approach has not identified specific SNVs or genes that could be followed up as potential drivers of treatment outcomes, we find genome-wide correlations that recapitulate previous epidemiological findings, such as the association of treatment resistance with poorer cognitive performance.^52,53^ Such polygenic overlaps validate our indirect approach for carrying out the GWAS of TRS in the absence of a single large-scale harmonized case-case sample. However, they should not be interpreted as proof of causality in either direction and do not account for confounders, such as sociodemographic indicators, medication adherence, or antipsychotic effects in treatment response.

### Implications

In revealing the existence of a common, heritable genetic signal for TRS, our study adds a new layer of evidence to the ongoing discussion of whether TRS is categorically distinct from treatment-responsive schizophrenia.^17^ Despite a large genetic correlation between both conditions, we show that PRS derived from them perform differently when attempting out-of-sample prediction of TRS, which suggests that the heterogeneous results previously obtained in PRS analyses might have been influenced by the presence of individuals with TRS in the generic schizophrenia training sample.^19^ While follow-up studies would likely require rich clinical data on large numbers of individuals with TRS and non-TRS to fully explore the implications of these results, they pose an important consideration for research seeking to understand whether the genetics of disorder susceptibility are the same as disease course in schizophrenia^2^ and whether treatment response can be influenced by the accumulation of genetic and clinical factors.^54,55^ Finally, while our results show that TRS is associated with a polygenic signal, the variance in TRS explained remains modest, and associated area under the curve values are small (eTable 3 in Supplement 1). Thus, polygenic scores tapping into this signal are thus unlikely to be of clinical utility in predicting treatment resistance, although their contribution to multifactorial predictive models (integrating rare variants, neuroimaging biomarkers, environmental exposures, demographic factors, and clinical measures) is an interesting avenue for future research.

### Limitations

This study has limitations. TRS is an underreported diagnosis, and while our research definition for this phenotype aligns with international criteria,^56^ we acknowledge that some individuals with treatment-resistant symptoms might still be present in the non-TRS data set, particularly in those samples where no ascertainment of TRS could be carried out. The effect of such misclassification would be akin to a reduction of effective sample size, reducing SNV discovery power and adding noise to GWAS effect size estimates.^57^ This further limits our power to detect differences in allelic associations through the interaction analysis, which is already only sensitive to relatively large differences in effect size between studies.^58^ Additionally, the interaction test does not model or addresses potential between-study heterogeneity. This precludes the use of more sophisticated statistical methods for downstream analyses, such as tissue-specific enrichment analyses or transcriptome-wide association studies.^59^

Imperfect phenotyping and potential misclassification might also have attenuated our results, and given the small total heritability detected for our TRS GWAS, we note that the magnitude of genetic correlations with other traits should be interpreted cautiously. Also, similar to what has been argued in the study of etiologically heterogeneous phenotypes through GWAS,^50,60^ the existence of significant genetic correlations and consistent polygenic association in independent samples reassures us that the common genetic variants we detect have (en masse) an association with TRS liability, even if our results cannot quantify the degree of contribution from nongenetic causes. In this regard, given that most of our analyses are based on European-based and UK-based samples, our conclusions might not be generalizable to non-European countries, where the diagnosis and treatment pathways of TRS might be influenced by racial and ethnic or cultural backgrounds.^61^

## Conclusions

In this GWAS, TRS had a small but detectable heritability associated with common risk alleles. Validation work, via the PRS method, showed that the contribution of these alleles were similar across incidence and prevalence samples. This is despite differences in cohort characteristics, and the use of drug prescription data as a proxy for TRS instead of quantitative metrics. Altogether, these results highlight the usefulness of well-controlled clinical phenotype data in psychiatric genetics to explore beyond diagnostic classifications and into treatment outcome and response to aid precision psychiatry.

## Notes

Supplement 1.
eMethods.
**eFigure 1.** Mirrored Manhattan plot of the 2 GWAS analysed with the TRS interaction procedure. **eFigure 2.** Q-Q plot of the TRS interaction GWAS. **eFigure 3.** PRS meta-analysis of CardiffCOGS and STRATA-G cohorts. **eTable 1.** Data sets included in the PGC non-TRS GWAS sample. **eTable 2. **Data sets included in the STRATA-G sample. **eTable 3.** Polygenic risk score analysis results. **eTable 4.** LD-Score and LD-Hub analyses of the TRS GWAS summary statistics.
eReferences.

Supplement 2. Nonauthor collaborators. Collaborators of the Genetics Workstream of the Schizophrenia Treatment Resistance and Therapeutic Advances (STRATA) Consortium and the Schizophrenia Working Group of the Psychiatric Genomics Consortium (PGC).
