# Supplementary material for: Interaction Testing and Polygenic Risk Scoring to Estimate the Association of Common Genetic Variants With Treatment Resistance in Schizophrenia
Source: JAMA Psychiatry. 2022 Jan 12;79(3):1–11. doi: 10.1001/jamapsychiatry.2021.3799 (PMC8756361; doi:10.1001/jamapsychiatry.2021.3799)
Supplement: Supplement 1. — eMethods. eFigure 1. Mirrored Manhattan plot of the 2 GWAS analysed with the TRS interaction procedure. eFigure 2. Q-Q plot of the TRS interaction GWAS. eFigure 3. PRS meta-analysis of CardiffCOGS and STRATA-G cohorts. eTable 1. Data sets included in the PGC non-TRS GWAS sample. eTable 2. Data sets included in the STRATA-G sample. eTable 3. Polygenic risk score analysis results. eTable 4. LD-Score and LD-Hub analyses of the TRS GWAS summary statistics. eReferences. [file jamapsychiatry-e213799-s001.pdf]

Supplemental Online Content

Pardiñas AF, Smart SE, Willcocks IR, et al; Genetics Workstream of the Schizophrenia Treatment Resistance and Therapeutic Advances (STRATA) Consortium; Schizophrenia Working Group of the Psychiatric Genomics Consortium (PGC). Interaction testing and polygenic risk scoring to estimate the association of common genetic variants with treatment resistance in schizophrenia. *JAMA Psychiatry*. Published online January 12, 2022. doi:10.1001/jamapsychiatry.2021.3799

eMethods.

eFigure 1. Mirrored Manhattan plot of the 2 GWAS analysed with the TRS interaction procedure

eFigure 2. Q-Q plot of the TRS interaction GWAS

eFigure 3. PRS meta-analysis of CardiffCOGS and STRATA-G cohorts

eTable 1. Data sets included in the PGC non-TRS GWAS sample

eTable 2. Data sets included in the STRATA-G sample

eTable 3. Polygenic risk score analysis results

eTable 4. LD-Score and LD-Hub analyses of the TRS GWAS summary statistics

eReferences.

This supplementary material has been provided by the authors to give readers additional information about their work.

© 2022 Pardiñas AF et al. *JAMA Psychiatry*.

eMethods.

CLOZUK and PGC data processing and GWAS

Due to the number of different datasets (45) and genotyping arrays (11) jointly involved in this analysis, processing of the TRS and non-TRS GWAS samples was done separately on data generated by the original studies. Imputed genotypes for the TRS analysis were those used by Pardiñas et al. (2018), which were inferred to be predominantly of UK genetic ancestry after principal component analysis (PCA) and ADMIXTURE estimation, and contain no detectable population outliers. Genotypes for the non-TRS analysis were obtained from Schizophrenia Working Group of the Psychiatric Genomics Consortium (2014), following the extensive curation process carried out during this work to discard population outliers and assess stratification.

Both of these imputations used the SHAPEIT/IMPUTE2 pipeline (Howie et al., 2012; Delaneau et al., 2013), though different versions of the 1000 Genomes reference panel were used in CLOZUK and PGC (Phase 3 and Phase 1 respectively). Given that the use of either reference has been found to result in similar imputation accuracies for SNPs with common (>5%) allele frequencies (1000 Genomes Project Consortium, 2015), we restricted to SNPs with a minor allele frequency (MAF) of 5% or higher in both datasets. We also excluded INDELs since their imputation performance tends to be lower than other common variants (Cirulli et al., 2014). Finally, the same post-imputation filters were applied to all remaining SNPs (INFO>0.6, Hardy Weinberg Equilibrium mid p-value > 10^{-6}).

In carrying out the GWAS of these samples, we followed the procedures outlined in the original studies from Pardiñas et al. (2018) and Schizophrenia Working Group of the Psychiatric Genomics Consortium (2014), including controlling for population stratification using covariates derived by PCA (Peloso and Lunetta, 2011). Combined results from the PGC non-TRS GWAS were meta-analysed using the fixed-effects procedure implemented in METAL v2011-03-25 (Willer et al., 2010). Only SNPs called in at least 20,000 combined samples were retained, and any strand-ambiguous markers (A/T, G/C) with MAF≥40% were discarded.

Comparison of the interaction test with the CC-GWAS method

To complement our analytic approach, we also investigated using the recently published CC-GWAS software (Peyrot and Price, 2020), intended for case/case designs similar to ours. However, the high genetic correlation between the TRS and non-TRS GWAS precluded us from using the CC-$GWAS_{OLS}$ or CC-$GWAS_{Exact}$ algorithms due to potential inflation of the false positive rate, with the only alternative being the lesser-powered CC-$GWAS_{Delta}$. Results of a CC-$GWAS_{Delta}$ analysis were closely aligned to our

© 2022 Pardiñas AF et al. *JAMA Psychiatry*.

interaction analysis (rg=0.992), returning the same λ and h^2 estimates to the third decimal place. Thus, for simplicity, we retained the test for interaction as our main genome-wide analysis.

© 2022 Pardiñas AF et al. *JAMA Psychiatry*.

CardiffCOGS genotyping and imputation

Genotypes from the CardiffCOGS samples were collected and curated as described in Pardiñas et al. (2018). Imputation of CardiffCOGS followed the procedure used for the CLOZUK GWAS samples which relied on the 1000 Genomes Phase 3 reference panel, and only SNPs passing a set of post-imputation quality control thresholds were retained (Genotype probability > 90%; INFO >0.8; Missingness < 5%; Hardy Weinberg Equilibrium p-value > 10^{-6}).

STRATA-G sample details

The participants included in STRATA-G are subsamples of participants from the studies listed below. Participants were included in STRATA-G if (1) ethical approval could be obtained to share data with the STRATA consortium, (2) blood, DNA, or genotype data was available to the STRATA-G researchers, and (3) participants had participated in a follow-up study, a minimum of 1 year after baseline. When additional criteria restricted which participants could be included in STRATA-G, we report the details of these below. We used a history of clozapine use to define our primary outcome variable of treatment-resistant schizophrenia.

AESOP (London, UK): The AESOP study (Aetiology and Ethnicity in Schizophrenia and Other Psychoses) is a multi-centre, naturalistic, prospective incidence and case-control study of first-episode psychosis, conducted initially over three years, from September 1997 to August 2000. The study sample comprises: all patients with an International Statistical Classification of Diseases and Related Health Problems, Tenth Revision (ICD-10) diagnosis of F10-F29 or F30-F33, aged 16-65 years, who presented to secondary and tertiary services within tightly defined catchment areas in south-east London, Nottingham, and Bristol (Dazzan et al., 2005; Fearon et al., 2006; Kirkbride et al., 2006; Morgan et al., 2006; Zimbron et al., 2014; Dean et al., 2018). All participants, in centres in southeast London and Nottingham (UK), were invited to take part in a follow-up study, at approximately 10 years after baseline (Morgan et al., 2014; Revier et al., 2015; Demjaha et al., 2017). Treatment resistance/non-resistance and history of clozapine use were determined by Dr Arsime Demjaha (Demjaha et al., 2017) and Dr Sophie Smart.

ESS (Prague, Czech Republic): The Early Stages of Schizophrenia (ESS) study is a hospital-based incidence study of first-episode schizophrenia, conducted initially over an unreported period of time. The study sample comprises: all patients with a ICD-10 diagnosis of F20 or F23, aged 18-35 years, with had less than 2 years of untreated psychosis, who were hospitalised in a large general psychiatry hospital that serves Prague and part of Central Bohemia regions (Melicher et al., 2015; Mikolas et al.,

© 2022 Pardiñas AF et al. *JAMA Psychiatry*.

2016; Spaniel et al., 2016; Kolenic et al., 2018). All participants were invited to take part in follow-up

© 2022 Pardiñas AF et al. *JAMA Psychiatry*.

studies, 1 year after baseline. Treatment resistance/non-resistance was determined by Dr Lina Homman.

EUGEI & BoFEP (Bologna, Italy): The Bologna data is from two studies. EUGEI (European Network of National Schizophrenia Networks Studying Gene-Environment Interactions) is a multi-centre, population-based incidence and case-sibling-control study of first-episode psychosis conducted initially, in Bologna, over a four-year period from January 2011 to December 2014. The study sample comprises: all patients with a ICD-10 diagnosis of F20-F33, aged 18-64 years, who presented to services within the catchment area (Jongsma et al., 2018). All participants, in Bologna, were invited totake part in a follow-up study, in 2016. The BoFEP study (Bologna FEP) is an ongoing, naturalistic, prospective incidence study of first-episode psychosis, conducted initially over an eight-year period from January 2002 and December 2009. The study sample comprises: all patients with a ICD-10 diagnosis of F10–F29 or F30–F33, aged 18-64 years, who presented to services within the defined catchment area in West Bologna. All participants were invited to take part in a follow-up study, 1 yearafter baseline (Tarricone et al., 2012). Treatment resistance/non-resistance, in both samples, was determined by Dr Lina Homman.

EUGEI Istanbul (Turkey): The Istanbul data is from an ongoing, hospital-based incidence study of first-episode schizophrenia, conducted in 1996. This study is sometimes known as the First-Episode Schizophrenia Follow-Up Project and a proportion of this sample was included in EUGEI. The study sample comprises: all patients with a DSM-IV diagnosis of schizophrenia, aged 15-45 years, who were experiencing an acute phase of their first psychotic episode and being treated as an inpatient (Üçok et al., 2004; Üçok et al., 2006; Üçok et al., 2011; Üçok et al., 2016). All participants were invited to takepart in follow up studies, 2+ years after baseline (Üçok et al., 2016). Treatment resistance/non- resistance was determined by Dr Alp Üçok.

EUGEI Paris (France): EUGEI is a multi-centre, population-based incidence and case-sibling-control of first-episode psychosis, conducted initially, in Créteil and Paris, over a two-year period from June 2012 to June 2014. The study sample comprises: all patients with a ICD-10 diagnosis of F20-F33, aged 18-64 years, who presented to services within the catchment area (Jongsma et al., 2018). All participants, in Créteil and Paris, were invited to take part in a follow-up study, in 2017. Treatment resistance/non-resistance was determined by Dr Andrei Szöke and Jean-Romain Richard.

GAP (London, UK): The GAP study (Genetics and Psychosis) is a population-based incidence and case-control study of first-episode psychosis, conducted initially over a three-year period from December 2005 to October 2010. The study sample comprises: all patients with a ICD-10 diagnosis of F20-F29 or

© 2022 Pardiñas AF et al. *JAMA Psychiatry*.

F30-F33, aged 18-65 years, who presented to secondary and tertiary services within tightly defined catchment areas in south-east London (Di Forti et al., 2009; Di Forti et al., 2015). The study exclusion criteria were evidence of 1) psychotic symptoms precipitated by an organic cause; 2) evidence of transient psychotic symptoms resulting from acute intoxication as defined by ICD-10; 3) moderate or severe learning disabilities as defined by ICD-10; or 4) head injury causing clinically significant loss of consciousness. Approximately 5 years after the first contact for psychosis, the follow-up data were extracted retrospectively using the electronic clinical records that are the primary clinical records keeping system within the Trust. This enables searching all clinical information, including correspondence, discharge letters and events, recorded throughout patients' journeys through the Trust (Lally et al., 2016a; Ajnakina et al., 2017). Treatment resistance/non-resistance was determined by Dr Olesya Ajnakina and Dr John Lally (Lally et al., 2016a).

NIFEPS & RGPI (Belfast, UK): The Belfast data is from two studies. The NIFEPS study (Northern Ireland First Episode Psychosis) is a naturalistic, prospective, incidence study, conducted initially over two years from January 2003 and December 2004. The study sample comprises: all patients with an Operational Criteria checklist for Psychotic Illness (OPCRIT) diagnosis of first-episode psychosis, aged 18–64 years, and living in Northern Ireland. All participants were invited to take part in follow-up studies, 1 year after baseline (Turkington et al., 2018) and approximately 13 years after baseline as part of the STRATA consortium. The RGPI study (Resources for Genomics, Ireland) is a multi-centre, population-based, incidence study of first episode of psychosis in 2007. The study sample comprises: all patients with a Diagnostic Statistical Manual IV (DSM-IV) diagnosis of schizophrenia, schizophreniform disorder, schizoaffective disorder or bipolar affective disorder with psychosis, aged 16+ years, who had Irish born grandparents, and who presented to psychiatric services in the region of the research centres (Casey and Corvin, 2008). All participants, recruited through psychiatric services in the region of Queen’s University, Belfast, were invited to take part in a follow-up study, at approximately 9 years after baseline as part of the STRATA consortium. Treatment resistance/non-resistance, in both samples, was determined by Dr Lina Homman.

PAFIP (Santander, Spain): The PAFIP study is an ongoing, naturalistic, prospective incidence study of first-episode psychosis, conducted from February 2001. The study sample comprises: all patients with an DSM-IV diagnosis of non-affective psychosis, aged 15+ years, who were referred from mental health services in the region of Cantabria. All participants were invited to take part in follow-up studies, 3+ years after baseline (Crespo-Facorro et al., 2007; Pelayo-Teran et al., 2008; Ayesa-Arriola et al., 2018; Setien-Suero et al., 2018). Treatment resistance/non-resistance was determined by Dr. Benedicto Crespo-Facorro and Dr. Javier Vázquez-Bourgon.

© 2022 Pardiñas AF et al. *JAMA Psychiatry*.

TIPP (Lausanne, Switzerland): The TIPP study (Treatment and Early Intervention in Psychosis Program) is an ongoing, naturalistic prospective study of early-onset psychosis, conducted from 2004. The study sample comprises: all patients who meet threshold criteria for psychosis (defined by the 'Psychosis threshold' subscale of the Comprehensive Assessment of At Risk Mental States (CAARMS) scale), aged 18-35 years, who reside in the Lausanne catchment area (Baumann et al., 2013a; Golay et al., 2016; Alameda et al., 2017). All participants enrolled in TIPP are invited to take part in follow up studies, lasting 3 years after baseline. A subsample of TIPP patients was included in STRATA-G: those that participated either in a neurobiological research study developed by Prof Kim Do (Baumann et al., 2013b), and/or were part of PsyMetab or Psyclin studies (Choong et al., 2008; Choong et al., 2013; Delacretaz et al., 2015; Quteineh et al., 2015; Vandenberghe et al., 2015). Treatment resistance/non-resistance, in both studies, was determined by Dr Romeo Restellini and Dr Luis Alameda.

TOP (Oslo, Norway): The TOP study (Thematic Organized Psychosis Research) is a naturalistic, prospective incidence and case-control study of first-episode psychosis, conducted initially over a four-year period from May 2003 to July 2007. The study sample comprises: all patients with a DSM-IV diagnosis of schizophrenia, schizophreniform disorder, schizoaffective disorder, psychosis not otherwise specified (NOS), delusional disorder, brief psychosis or major affective disorder with mood incongruent psychotic symptoms, aged 18-65 years, within 1 year of the start of their first adequate treatment with antipsychotic medication, who presented to outpatient and inpatient services within four University Hospitals in Oslo (Faerden et al., 2008; Athanasiu et al., 2010). All participants were invited to take part in a follow-up study, approximately 1 year after baseline (Faerden et al., 2013; Lange et al., 2014; Lyngstad et al., 2018). Treatment resistance/non-resistance was determined by Dr Carmen Simonsen and Professor Ingrid Melle.

West London (London, UK): The West London Longitudinal First-Episode Psychosis Study a naturalistic, prospective incidence study of first-episode psychosis, conducted from 1998 to 2008. The study sample comprises: all patients with an DSM-IV diagnosis of psychosis, aged 16-50 years, who were presenting with a psychotic illness for the first time and had been receiving antipsychotic medication for less than 12 weeks. All participants were invited to take part in follow-up studies, 1+ years after baseline (Huddy et al., 2007; Gutierrez-Galve et al., 2010; Huddy et al., 2013; Gutierrez-Galve et al., 2015). Treatment resistance/non-resistance was determined by Dr Sophie Smart.

STRATA-G genotyping and imputation

All the STRATA-G individuals were genotyped using Illumina platforms as a part of their respective studies; array details are provided in **Supplementary Table 2**. Within each dataset, basic genotypic

© 2022 Pardiñas AF et al. *JAMA Psychiatry*.

quality control (QC) was performed using PLINK v1.9 (Chang et al., 2015) following standard procedures (Anderson et al., 2010), and allowing for 5% of missing data at the marker and individual level. Datasets were then merged into two batches by the similarity of their array content, and all non-overlapping markers were discarded. Relatedness between individuals was assessed in a merged dataset consisting of 203,813 SNPs common between both batches, using the PC-Relate approach (Conomos et al., 2016). A random member of each related pair (ϕ>0.2) was selected for removal in further analyses, prioritising retaining TRS individuals.

Imputation was performed on each batch separately using the Minimac4 algorithm as provided by the Michigan Imputation Server (Das et al., 2016) and HRC reference panel, which has similar accuracy for common variation as the 1000 Genomes Phase 3 panel used in CardiffCOGS (McCarthy et al., 2016). After this process, imputed dosages were converted to best-guess genotype calls for use in polygenic scoring (Genotype probability > 90%; INFO > 0.8, MAF > 1%, HWE mid p-value > 10^{-4}). For their use in association testing, principal components were generated from the post-imputed data using markers in relative linkage equilibrium (r^2<0.2) and the PC-AiR algorithm (Conomos et al., 2015).

Polygenic validation analyses

To avoid the known bias in PRS analysis when samples are present in both training and testing sets, we ensured that no samples from either CardiffCOGS or STRATA-G were included in any discovery GWAS analyses, and that any samples related to either of these cohorts (relatedness coefficient ϕ>0.2) were removed. All summary statistics were curated by retaining only non-ambiguous SNPs outside of long-range LD regions (Price et al., 2008) that had a MAF of 10% or higher and INFO ≥0.9.

In PRSice-2, p-value thresholds for the computed scores were set at 8 different intervals ($p<10^{-5}$, $p<10^{-4}$, p<0.001, p<0.05, p<0.01, p<0.1, p<0.5, p<1). Finally, a score was also generated via the PRS-CS software (Ge et al., 2019), which estimates PRS using all SNPs in a Bayesian framework. The UK-Biobank LD reference provided with PRS-CS was used for all computations. The global shrinkage parameter was set to $\phi=1x10^{-4}$ for the TRS interaction and $\phi=1x10^{-2}$ for the CLOZUK and PGC GWAS, reflecting the differential polygenicity of these analyses, as recommended by the software manual.

Association of the polygenic scores with the TR/non-TRS phenotype was calculated using logistic models with sex and the first 5 genotypic principal components (PCs) as covariates, in order to control for population stratification (Peloso and Lunetta, 2011). Given the presence of multiple ancestries in STRATA-G samples, we employed a previously described model to classify each sample into seven broadly defined biogeographical population groups using ancestry-informative markers (AIMs; Legge et al., 2019). We then used the underlying discriminant functions of this model to compute six

© 2022 Pardiñas AF et al. *JAMA Psychiatry*.

variables reflecting the probability of each sample being classified as “European”, “South West Asian”,“East Asian”, “Subsaharan African” or “North African”; and included these as covariates in all PRS analyses involving STRATA-G. To assess variance explained by the PRS metrics, Nagelkerke’s R^2 valuesfor each PRS logistic regression were calculated on the liability scale (Lee et al., 2012), assuming a 30% population prevalence for TRS in CardiffCOGS (Lally et al., 2016b) and 15% prevalence as a conservative estimate for STRATA-G (Kanahara et al., 2018; Siskind et al., 2021).

Finally, for the STRATA-G analyses, since this cohort was imputed in two independent batches, PRS association tests and their summary statistics were computed separately within each STRATA-G imputation batch (**Supplementary Table 2**), with batch 1 consisting of 466 individuals (46 TRS; 420 non-TRS) and batch 2 consisting of 103 individuals (25 TRS; 72 non-TRS). Effect size statistics reported in **Supplementary Table 3** were derived from the original betas and standard errors using fixed-effect meta-analysis with inverse variance weights. Pooled liability-scale R^2 were computed with the method of Harel (2009). Meta-analytic AUC values were computed with the random-effects method of Debray et al. (2017). All these computations were performed with functions from the R package “*metafor*" (Viechtbauer, 2010). An analogous meta-analysis was also carried out using the association summary statistics from CardiffCOGS and both STRATA-G batches (**Supplementary Figure 3**).

© 2022 Pardiñas AF et al. *JAMA Psychiatry*.

PGC TRS rating system

Cohort	Definition of assessed phenotypes	
	TRS	Non-TRS
aber - Aberdeen, UK	Ever treated with clozapine.	Never treated with clozapine.
asrb - Australia	Treated with clozapine at the time of the diagnostic assessment.	Not treated with clozapine at the time of the diagnostic assessment.
boco - Bonn/Mannheim, Germany	History of clozapine treatment at the time of interview.	No (explicit) disclosure of history of clozapine treatment during interview or in provided patient records.
denm - Denmark	Diagnosed with incident schizophrenia in Danish national registry data (1/Jan/1996 - 31/Dec/2010) and with recorded clozapine initiation.	Diagnosed with incident schizophrenia in Danish national registry data (1/Jan/1996 - 31/Dec/2010) but no recorded clozapine initiation.
dubl - Ireland	Ever treated with clozapine.	Never treated with clozapine.
irwt - Ireland (WTCCC2)	Ever treated with clozapine.	Never treated with clozapine.
munc - Munich, Germany	Ever treated with clozapine.	Never treated with clozapine.
port - Portugal	Negatively rated for OPCRIT item 89 ("psychotic symptoms respond to neuroleptics"; McGuffin et al., 1991).	Positively rated for OPCRIT item 89.
swe[1,5,6]/s234 - Sweden	Redeemed a clozapine prescription between the start of the Swedish drug register and the end of data collection (1/Jul/2005 - 31/Dec/2013)	Did not redeem a clozapine prescription in the same timeframe (1/Jul/2005 - 31/Dec/2013)
top8 - Oslo, Norway	Treated with or considered for clozapine during the follow up period.	Never treated with or considered for clozapine.
uclo - London, UK	Ever treated with clozapine.	Never treated with clozapine.

© 2022 Pardiñas AF et al. *JAMA Psychiatry*.

STRATA-G TRS rating system

Cohort	Definition of assessed phenotypes	
	TRS	Non-TRS
AESOP (London, UK)	Treated with clozapine during the follow up period.	A state, of at least 6 months' duration, in which no symptoms or only symptoms of mild severity, not interfering with daily functioning, were experienced (Andreasen et al., 2005)
ESS (Prague, Czech Republic)	Treated with or considered for clozapine during the follow up period.	Never treated with or considered for clozapine.
EUGEI & BoFEP (Bologna, Italy)	Treated with or considered for clozapine during the follow up period.	Never treated with or considered for clozapine.
EUGEI Istanbul (Turkey)	Treated with or considered for clozapine during the follow up period.	Never treated with or considered for clozapine.
EUGEI Paris (France)	Treated with or considered for clozapine during the follow up period.	Never treated with or considered for clozapine.
GAP (London, UK)	Treated with or considered for clozapine during the follow up period. Excluded those who were intolerant of antipsychotic medications or those who self-discontinued medication.	Never treated with or considered for clozapine.
NIFEPS & RGPI (Belfast, UK)	Treated with or considered for clozapine during the follow up period.	Never treated with or considered for clozapine.
PAFIP (Santander, Spain)	Treated with or considered for clozapine during the follow up period.	Never treated with or considered for clozapine.
TIPP (Switzerland)	Treated with or considered for clozapine during the follow up period. Excluded those with poor compliance and who met criteria for symptom severity.	Never treated with or considered for clozapine.
TOP (Oslo, Norway)	Treated with or considered for clozapine during the follow up period.	Never treated with or considered for clozapine.
West London (London, UK)	Treated with clozapine at the time of a follow up interview.	Never treated with clozapine at the time of a follow up interview.

© 2022 Pardiñas AF et al. *JAMA Psychiatry*.

Members of the Schizophrenia Working Group of the Psychiatric Genomics Consortium

Stephan Ripke[1,2], Benjamin M. Neale[1,2,3,4], Aiden Corvin[5], James T. R. Walters[6], Kai-How Farh[1], Peter A. Holmans[6,7], Phil Lee[1,2,4], Brendan Bulik-Sullivan[1,2], David A. Collier[8,9], Hailiang Huang[1,3], Tune H. Pers[3,10,11], Ingrid Agartz[12,13,14], Esben Agerbo[15,16,17], Margot Albus[18], Madeline Alexander[19], Farooq Amin[20,21], Silviu A. Bacanu[22], Martin Begemann[23], Richard A Belliveau Jr[2], Judit Bene[24,25], Sarah E. Bergen [2,26], Elizabeth Bevilacqua[2], Tim B Bigdeli [22], Donald W. Black[27], Richard Bruggeman[28], Nancy G. Buccola[29], Randy L. Buckner[30,31,32], William Byerley[33], Wiepke Cahn[34], Guiqing Cai[35,36], Murray J. Cairns[39,120,170], Dominique Campion[37], Rita M. Cantor[38], Vaughan J. Carr[39,40], Noa Carrera[6], Stanley V. Catts[39,41], Kimberly D. Chambert[2], Raymond C. K. Chan[42], Ronald Y. L. Chen[43], Eric Y. H. Chen[43,44], Wei Cheng[45], Eric F. C. Cheung[46], Siow Ann Chong[47], C. Robert Cloninger[48], David Cohen[49], Nadine Cohen[50], Paul Cormican[5], Nick Craddock[6,7], James J. Crowley[51], David Curtis[52,53], Michael Davidson[54], Kenneth L. Davis[36], Franziska Degenhardt[55,56], Jurgen Del Favero[57], Lynn E. DeLisi[128,129] , Ditte Demontis[17,58,59], Dimitris Dikeos[60], Timothy Dinan[61], Srdjan Djurovic[14,62], Gary Donohoe[5,63], Elodie Drapeau[36], Jubao Duan[64,65], Frank Dudbridge[66], Naser Durmishi[67], Peter Eichhammer[68], Johan Eriksson[69,70,71], Valentina Escott-Price[6], Laurent Essioux[72], Ayman H. Fanous[73,74,75,76], Martilias S. Farrell[51], Josef Frank[77], Lude Franke[78], Robert Freedman[79], Nelson B. Freimer[80], Marion Friedl[81], Joseph I. Friedman[36], Menachem Fromer[1,2,4,82], Giulio Genovese[2], Lyudmila Georgieva[6], Elliot S. Gershon[209], Ina Giegling[81,83], Paola Giusti-Rodríguez[51], Stephanie Godard[84], Jacqueline I. Goldstein[1,3], Vera Golimbet[85], Srihari Gopal[86], Jacob Gratten[87], Lieuwe de Haan[88], Christian Hammer[23], Marian L. Hamshere[6], Mark Hansen[89], Thomas Hansen[17,90], Vahram Haroutunian[36,91,92], Annette M. Hartmann[81], Frans A. Henskens[39,93,94], Stefan Herms[55,56,95], Joel N. Hirschhorn[3,11,96], Per Hoffmann[55,56,95], Andrea Hofman[55,56], Mads V. Hollegaard[97], David M. Hougaard[97], Masashi Ikeda[98], Inge Joa[99], Antonio Julià[100], René S. Kahn[34], Luba Kalaydjieva[101,102], Sena Karachanak-Yankova[103], Juha Karjalainen[78], David Kavanagh[6], Matthew C. Keller[104], Brian J. Kelly[120], James L. Kennedy[105,106,107], Andrey Khrunin[108], Yunjung Kim[51], Janis Klovins[109], James A. Knowles[110], Bettina Konte[81], Vaidutis Kucinskas[111], Zita Ausrele Kucinskiene[111], Hana Kuzelova-Ptackova[112], Anna K. Kähler[26], Claudine Laurent[19,113], Jimmy Lee Chee Keong[47,114], S. Hong Lee[87], Sophie E. Legge[6], Bernard Lerer[115], Miaoxin Li[43,44,116] Tao Li[117], Kung-Yee Liang[118], Jeffrey Lieberman[119], Svetlana Limborska[108], Carmel M. Loughland[39,120], Jan Lubinski[121], Jouko Lönnqvist[122], Milan Macek Jr[112], Patrik K. E. Magnusson[26], Brion S. Maher[123], Wolfgang Maier[124], Jacques Mallet[125], Sara Marsal[100], Manuel Mattheisen[17,58,59,126], Morten Mattingsdal[14,127], Robert W. McCarley[128,129], Colm McDonald[130], Andrew M. McIntosh[131,132], Sandra Meier[77], Carin J. Meijer[88], Bela Melegh[24,25], Ingrid Melle[14,133], Raquelle I. Mesholam-Gately[128,134], Andres Metspalu[135], Patricia T. Michie[39,136], Lili Milani[135], Vihra Milanova[137], Younes Mokrab[8], Derek W. Morris[5,63], Ole Mors[17,58,138], Kieran C. Murphy[139], Robin M. Murray[140], Inez Myin-Germeys[141], Bertram Müller-Myhsok[142,143,144], Mari Nelis[135], Igor Nenadic[145], Deborah A. Nertney[146], Gerald Nestadt[147], Kristin K. Nicodemus[148], Liene Nikitina-Zake[109], Laura Nisenbaum[149], Annelie Nordin[150], Eadbhard O'Callaghan[151], Colm O'Dushlaine[2], F. Anthony O'Neill[152], Sang-Yun Oh[153], Ann Olincy[79], Line Olsen[17,90], Jim Van Os[141,154], Psychosis Endophenotypes International Consortium[155], Christos Pantelis[39,156], George N. Papadimitriou[60], Sergi Papiol[23], Elena Parkhomenko[36], Michele T. Pato[110], Tiina Paunio[157,158], Milica Pejovic-Milovancevic[159], Diana O. Perkins[160], Olli Pietiläinen[158,161], Jonathan Pimm[53], Andrew J. Pocklington[6], John Powell[140], Alkes Price[3],[162], Ann E. Pulver[147], Shaun M. Purcell[82], Digby Quested[163], Henrik B. Rasmussen[17,90], Abraham Reichenberg[36], Mark A. Reimers[164], Alexander L. Richards[6], Joshua L. Roffman[30,32], Panos Roussos[82,165], Douglas M. Ruderfer[6,82], Veikko Salomaa[71], Alan R. Sanders[64,65], Ulrich Schall[39,120], Christian R. Schubert[166], Thomas G. Schulze[77,167], Sibylle G. Schwab[168], Edward M. Scolnick[2], Rodney J. Scott[39,169,170], Larry J. Seidman[128,134], Jianxin Shi[171], Engilbert Sigurdsson[172], Teimuraz Silagadze[173], Jeremy M. Silverman[36,174], Kang Sim[47], Petr Slominsky[108], Jordan W. Smoller[2,4], Hon-Cheong So[43], Chris C. A. Spencer[175], Eli A. Stahl[3,82], Hreinn Stefansson[176], Stacy Steinberg[176], Elisabeth Stogmann[177], Richard E. Straub[178], Eric Strengman[179,34], Jana Strohmaier[77], T. Scott Stroup[119], Mythily Subramaniam[47], Jaana

Suvisaari[122], Dragan M. Svrakic[48], Jin P. Szatkiewicz[51], Erik Söderman[12], Srinivas Thirumalai[180], Draga Toncheva[103], Paul A. Tooney[39,120,170] , Sarah Tosato[181], Juha Veijola[182,183], John Waddington[184], Dermot Walsh[185], Dai Wang[86], Qiang Wang[117], Bradley T. Webb[22], Mark Weiser[54], Dieter B. Wildenauer[186], Nigel M. Williams[6], Stephanie Williams[51], Stephanie H. Witt[77], Aaron R. Wolen[164], Emily H. M. Wong[43], Brandon K. Wormley[22], Sathish Periyasamy [146], Brian Kelly [39,170], Hualin Simon Xi[187], Clement C. Zai[105,106], Xuebin Zheng[188], Fritz Zimprich[177], Naomi R. Wray[87], Kari Stefansson[176], Peter M. Visscher[87], Wellcome Trust Case-Control Consortium 2[189], Rolf Adolfsson[150], Ole A. Andreassen[14,133], Douglas H. R. Blackwood[132], Elvira Bramon[190], Joseph D. Buxbaum[35,36,91,191], Anders D. Børglum[17,58,59,138], Sven Cichon[55,56,95,192], Ariel Darvasi[193], Enrico Domenici[194], Hannelore Ehrenreich[23], Tõnu Esko[3,11,96,135], Pablo V. Gejman[64,65], Michael Gill[5], Hugh Gurling[53], Christina M. Hultman[26], Nakao Iwata[98], Assen V. Jablensky[39,102,186,195], Erik G. Jönsson[12,14], Kenneth S. Kendler[196], George Kirov[6], Jo Knight[105,106,107], Todd Lencz[197,198,199], Douglas F. Levinson[19], Qingqin S. Li[86], Jianjun Liu[188,200], Anil K. Malhotra[197,198,199], Steven A. McCarroll[2,96], Andrew McQuillin[53], Jennifer L. Moran[2], Preben B. Mortensen[15,16,17], Bryan J. Mowry[87,201], Markus M. Nöthen[55,56], Roel A. Ophoff[38,80,34], Michael J. Owen[6,7], Aarno Palotie[2,4,161], Carlos N. Pato[110], Tracey L. Petryshen[2,128,202], Danielle Posthuma[203,204,205], Marcella Rietschel[77], Brien P. Riley[196], Dan Rujescu[81,83], Pak C. Sham[43,44,116] Pamela Sklar[82,91,165], David St Clair[206], Daniel R. Weinberger[178,207], Jens R. Wendland[166], Thomas Werge[17,90,208], Mark J. Daly[1,2,3], Patrick F. Sullivan[26,51,160] & Michael C. O'Donovan[6,7]

Affiliations: [1]Analytic and Translational Genetics Unit, Massachusetts General Hospital, Boston, Massachusetts 02114, USA. [2]Stanley Center for Psychiatric Research, Broad Institute of MIT and Harvard, Cambridge, Massachusetts 02142, USA. [3]Medical and Population Genetics Program, Broad Institute of MIT and Harvard, Cambridge, Massachusetts 02142, USA. [4]Psychiatric and Neurodevelopmental Genetics Unit, Massachusetts General Hospital, Boston, Massachusetts 02114, USA. [5]Neuropsychiatric Genetics Research Group, Department of Psychiatry, Trinity College Dublin, Dublin 8, Ireland. [6]MRC Centre for Neuropsychiatric Genetics and Genomics, Institute of Psychological Medicine and Clinical Neurosciences, School of Medicine, Cardiff University, Cardiff, CF24 4HQ, UK. [7]National Centre for Mental Health, Cardiff University, Cardiff, CF24 4HQ, UK. [8]Eli Lilly and Company Limited, Erl Wood Manor, Sunninghill Road, Windlesham, Surrey, GU20 6PH, UK. [9]Social, Genetic and Developmental Psychiatry Centre, Institute of Psychiatry, King's College London, London, SE5 8AF, UK. [10]Center for Biological Sequence Analysis, Department of Systems Biology, Technical University of Denmark, DK-2800, Denmark. [11]Division of Endocrinology and Center for Basic and Translational Obesity Research, Boston Children's Hospital, Boston, Massachusetts, 02115USA. [12]Department of Clinical Neuroscience, Psychiatry Section, Karolinska Institutet, SE-17176 Stockholm, Sweden. [13]Department of Psychiatry, Diakonhjemmet Hospital, 0319 Oslo, Norway. [14]NORMENT, KG Jebsen Centre for Psychosis Research, Institute of Clinical Medicine, University of Oslo, 0424Oslo, Norway. [15]Centre for Integrative Register-based Research, CIRRAU, Aarhus University, DK-8210 Aarhus, Denmark. [16]National Centre for Register-based Research, Aarhus University, DK-8210 Aarhus, Denmark. [17]The Lundbeck Foundation Initiative for Integrative Psychiatric Research, iPSYCH, Denmark. [18]State Mental Hospital, 85540 Haar, Germany. [19]Department of Psychiatry and Behavioral Sciences, Stanford University, Stanford, California 94305, USA. [20]Department of Psychiatry and Behavioral Sciences, Atlanta Veterans Affairs Medical Center, Atlanta, Georgia 30033, USA. [21]Department of Psychiatry and Behavioral Sciences, Emory University, Atlanta Georgia 30322, USA. [22]Virginia Institute for Psychiatric and Behavioral Genetics, Department of Psychiatry, Virginia Commonwealth University, Richmond, Virginia 23298, USA. [23]Clinical Neuroscience, Max Planck Institute of Experimental Medicine, Göttingen 37075, Germany. [24]Department of Medical Genetics, University of Pécs, Pécs H-7624, Hungary. [25]Szentagothai Research Center, University of Pécs, Pécs H-7624, Hungary. [26]Department of Medical Epidemiology and Biostatistics, Karolinska Institutet, Stockholm SE-17177, Sweden. [27]Department of Psychiatry, University of Iowa Carver College of Medicine, Iowa City, Iowa 52242, USA. [28]University Medical Center Groningen, Department of Psychiatry, University of Groningen NL-9700 RB, The Netherlands. [29]School of Nursing, Louisiana State University Health

© 2022 Pardiñas AF et al. *JAMA Psychiatry*.

Sciences Center, New Orleans, Louisiana 70112, USA. [30]Athinoula A. Martinos Center, Massachusetts General Hospital, Boston, Massachusetts 02129, USA. [31]Center for Brain Science, Harvard University, Cambridge, Massachusetts, 02138 USA. [32]Department of Psychiatry, Massachusetts General Hospital, Boston, Massachusetts, 02114 USA. [33]Department of Psychiatry, University of California at San Francisco, San Francisco, California, 94143 USA. [34]University Medical Center Utrecht, Department of Psychiatry, Rudolf Magnus Institute of Neuroscience, 3584 Utrecht, The Netherlands. [35]Department of Human Genetics, Icahn School of Medicine at Mount Sinai, New York, New York 10029 USA. [36]Department of Psychiatry, Icahn School of Medicine at Mount Sinai, New York, New York 10029 USA. [37]Centre Hospitalier du Rouvray and INSERM U1079 Faculty of Medicine, 76301 Rouen, France. [38]Department of Human Genetics, David Geffen School of Medicine, University of California, Los Angeles, California 90095, USA. [39]Schizophrenia Research Institute, SydneyNSW 2010, Australia. [40]School of Psychiatry, University of New South Wales, Sydney NSW 2031, Australia. [41]Royal Brisbane and Women's Hospital, University of Queensland, Brisbane, St Lucia QLD 4072, Australia. [42]Institute of Psychology, Chinese Academy of Science, Beijing 100101, China. [43]Department of Psychiatry, Li Ka Shing Faculty of Medicine, The University of Hong Kong, Hong Kong, China. [44]State Key Laboratory for Brain and Cognitive Sciences, Li Ka Shing Faculty of Medicine, The University of Hong Kong, Hong Kong, China. [45]Department of Computer Science, University of North Carolina, Chapel Hill, North Carolina 27514, USA. [46]Castle Peak Hospital, Hong Kong, China. [47]Institute of Mental Health, Singapore 539747, Singapore. [48]Department of Psychiatry, Washington University, St. Louis, Missouri 63110, USA. [49]Department of Child and Adolescent Psychiatry, Assistance Publique Hopitaux de Paris, Pierre and Marie Curie Faculty of Medicine and Institute for Intelligent Systems and Robotics, Paris, 75013, France. [50] Blue Note Biosciences, Princeton, New Jersey 08540, USA [51]Department of Genetics, University of North Carolina, Chapel Hill, North Carolina 27599-7264, USA. [52]Department of Psychological Medicine, Queen Mary University of London, London E1 1BB, UK. [53]Molecular Psychiatry Laboratory, Division of Psychiatry, University College London, London WC1E 6JJ, UK. [54]Sheba Medical Center, Tel Hashomer 52621, Israel. [55]Department of Genomics, Life and Brain Center, D-53127 Bonn, Germany. [56]Institute of Human Genetics, University of Bonn, D-53127 Bonn, Germany. [57]Applied Molecular Genomics Unit, VIB Department of Molecular Genetics, University of Antwerp, B-2610 Antwerp, Belgium. [58]Centre for Integrative Sequencing, iSEQ, Aarhus University, DK-8000 Aarhus C, Denmark. [59]Department of Biomedicine, Aarhus University, DK-8000 Aarhus C, Denmark. [60]First Department of Psychiatry, University of Athens Medical School, Athens 11528, Greece. [61]Department of Psychiatry, University College Cork, Co. Cork, Ireland. [62]Department of Medical Genetics, Oslo University Hospital, 0424 Oslo, Norway. [63]Cognitive Genetics and Therapy Group, School of Psychology and Discipline of Biochemistry, National University of Ireland Galway, Co. Galway, Ireland. [64]Department of Psychiatry and Behavioral Neuroscience, University of Chicago, Chicago, Illinois 60637, USA. [65]Department of Psychiatry and Behavioral Sciences, NorthShore University HealthSystem, Evanston, Illinois 60201, USA. [66]Department of Non-Communicable Disease Epidemiology, London School of Hygiene and Tropical Medicine, London WC1E 7HT, UK. [67]Department of Child and Adolescent Psychiatry, University Clinic of Psychiatry, Skopje 1000, Republic of Macedonia. [68]Department of Psychiatry, University of Regensburg, 93053 Regensburg, Germany. [69]Department of General Practice, Helsinki University Central Hospital, University of Helsinki P.O. Box 20, Tukholmankatu 8 B, FI-00014, Helsinki, Finland [70]Folkhälsan Research Center, Helsinki, Finland, Biomedicum Helsinki 1, Haartmaninkatu 8, FI-00290, Helsinki, Finland.[71]National Institute for Health and Welfare, P.O. BOX 30, FI-00271 Helsinki, Finland.[72]Translational Technologies and Bioinformatics, Pharma Research and Early Development, F. Hoffman-La Roche, CH-4070 Basel, Switzerland. [73]Department of Psychiatry, Georgetown University School of Medicine, Washington DC 20057, USA. [74]Department of Psychiatry, Keck School of Medicine of the University of Southern California, Los Angeles, California 90033, USA. [75]Department of Psychiatry, Virginia Commonwealth University School of Medicine, Richmond, Virginia 23298, USA. [76]Mental Health Service Line, Washington VA Medical Center, Washington DC 20422, USA. [77]Department of Genetic Epidemiology in Psychiatry, Central Institute of Mental Health, Medical Faculty Mannheim, University of Heidelberg, Heidelberg , D-68159

© 2022 Pardiñas AF et al. *JAMA Psychiatry*.

Mannheim, Germany. [78]Department of Genetics, University of Groningen, University Medical Centre Groningen, 9700 RB Groningen, The Netherlands. [79]Department of Psychiatry, University of Colorado Denver, Aurora, Colorado 80045, USA. [80]Center for Neurobehavioral Genetics, Semel Institute for Neuroscience and Human Behavior, University of California, Los Angeles, California 90095, USA. [81]Department of Psychiatry, University of Halle, 06112 Halle, Germany. [82]Division of Psychiatric Genomics, Department of Psychiatry, Icahn School of Medicine at Mount Sinai, New York, New York 10029, USA. [83]Department of Psychiatry, University of Munich, 80336, Munich, Germany. [84]Departments of Psychiatry and Human and Molecular Genetics, INSERM, Institut de Myologie, Hôpital de la Pitiè-Salpêtrière, Paris, 75013, France. [85]Mental Health Research Centre, Russian Academy of Medical Sciences, 115522 Moscow, Russia. [86]Neuroscience Therapeutic Area, Janssen Research and Development, Raritan, New Jersey 08869, USA. [87]Queensland Brain Institute, The University of Queensland, Brisbane, Queensland, QLD 4072, Australia. [88]Academic Medical Centre University of Amsterdam, Department of Psychiatry, 1105 AZ Amsterdam, The Netherlands. [89]Illumina, La Jolla, California, California 92122,USA. [90]Institute of Biological Psychiatry, Mental Health Centre Sct. Hans, Mental Health Services Copenhagen, DK-4000, Denmark. [91]Friedman Brain Institute, Icahn School of Medicine at Mount Sinai, New York, New York 10029, USA. [92]J. J. Peters VA Medical Center, Bronx, New York, New York 10468, USA. [93]Priority Research Centre for Health Behaviour, University of Newcastle, Newcastle NSW 2308, Australia. [94]School of Electrical Engineering and Computer Science, University of Newcastle, Newcastle NSW 2308, Australia. [95]Division of Medical Genetics, Department of Biomedicine, University of Basel, Basel, CH-4058, Switzerland. [96]Department of Genetics, Harvard Medical School, Boston, Massachusetts 02115, USA. [97]Section of Neonatal Screening and Hormones, Department of Clinical Biochemistry, Immunology and Genetics, Statens Serum Institut, Copenhagen, DK-2300, Denmark. [98]Department of Psychiatry, Fujita Health University School of Medicine, Toyoake, Aichi, 470-1192, Japan. [99]Regional Centre for Clinical Research in Psychosis, Department of Psychiatry, Stavanger University Hospital, 4011 Stavanger, Norway. [100]Rheumatology Research Group, Vall d'Hebron Research Institute, Barcelona, 08035, Spain. [101]Centre for Medical Research, The University of Western Australia, Perth, WA 6009, Australia. [102]ThePerkins Institute for Medical Research, The University of Western Australia, Perth, WA 6009, Australia.[103]Department of Medical Genetics, Medical University, Sofia1431, Bulgaria. [104]Department of Psychology, University of Colorado Boulder, Boulder, Colorado 80309, USA. [105]Campbell Family Mental Health Research Institute, Centre for Addiction and Mental Health, Toronto, Ontario, M5T 1R8, Canada. [106]Department of Psychiatry, University of Toronto, Toronto, Ontario, M5T 1R8, Canada. [107]Institute of Medical Science, University of Toronto, Toronto, Ontario, M5S 1A8, Canada. [108]Institute of Molecular Genetics, Russian Academy of Sciences, Moscow123182, Russia. [109]Latvian Biomedical Research and Study Centre, Riga, LV-1067, Latvia. [110]Department of Psychiatry and Zilkha Neurogenetics Institute, Keck School of Medicine at University of Southern California, Los Angeles, California 90089, USA. [111]Faculty of Medicine, Vilnius University, LT-01513 Vilnius, Lithuania. [112] Department of Biology and Medical Genetics, 2nd Faculty of Medicine and University Hospital Motol, 150 06 Prague, Czech Republic. [113]Department of Child and Adolescent Psychiatry, Pierre and Marie Curie Faculty of Medicine, Paris 75013, France. [114]Duke-NUS Graduate Medical School, Singapore 169857, Singapore. [115]Department of Psychiatry, Hadassah-Hebrew University Medical Center, Jerusalem 91120, Israel. [116]Centre for Genomic Sciences, The University of Hong Kong, Hong Kong, China.[117]Mental Health Centre and Psychiatric Laboratory, West China Hospital, Sichuan University, Chengdu, 610041, Sichuan, China. [118]Department of Biostatistics, Johns Hopkins University Bloomberg School of Public Health, Baltimore, Maryland 21205, USA. [119]Department of Psychiatry, Columbia University, New York, New York 10032, USA. [120]Priority Centre for Translational Neuroscience and Mental Health, University of Newcastle, Newcastle NSW 2300, Australia.[121]Department of Genetics and Pathology, International Hereditary Cancer Center, Pomeranian Medical University in Szczecin, 70-453 Szczecin, Poland. [122]Department of Mental Health and Substance Abuse Services; National Institute for Health and Welfare, P.O. BOX 30, FI-00271 Helsinki, Finland [123]Department of Mental Health, Bloomberg School of Public Health, Johns Hopkins University, Baltimore, Maryland 21205, USA. [124]Department of Psychiatry, University of Bonn, D-53127

© 2022 Pardiñas AF et al. *JAMA Psychiatry*.

Bonn, Germany. [125]Centre National de la Recherche Scientifique, Laboratoire de Génétique Moléculaire de la Neurotransmission et des Processus Neurodégénératifs, Hôpital de la Pitié Salpêtrière, 75013, Paris, France. [126]Department of Genomics Mathematics, University of Bonn, D-53127 Bonn, Germany. [127]Research Unit, Sørlandet Hospital, 4604 Kristiansand, Norway. [128]Department of Psychiatry, Harvard Medical School, Boston, Massachusetts 02115, USA. [129]VA Boston Health Care System, Brockton, Massachusetts 02301, USA. [130]Department of Psychiatry, National University of Ireland Galway, Co. Galway, Ireland. [131]Centre for Cognitive Ageing and Cognitive Epidemiology, University of Edinburgh, Edinburgh EH16 4SB, UK. [132]Division of Psychiatry, University of Edinburgh, Edinburgh EH16 4SB, UK. [133]Division of Mental Health and Addiction, Oslo University Hospital, 0424 Oslo, Norway. [134]Massachusetts Mental Health Center Public Psychiatry Division of the Beth Israel Deaconess Medical Center, Boston, Massachusetts 02114, USA. [135]Estonian Genome Center, University of Tartu, Tartu 50090, Estonia. [136]School of Psychology, University of Newcastle, Newcastle NSW 2308, Australia. [137]First Psychiatric Clinic, Medical University, Sofia 1431, Bulgaria. [138]Department P, Aarhus University Hospital, DK-8240 Risskov, Denmark. [139]Department of Psychiatry, Royal College of Surgeons in Ireland, Dublin 2, Ireland. [140]King's College London, London SE5 8AF, UK. [141]Maastricht University Medical Centre, South Limburg Mental Health Research and Teaching Network, EURON, 6229 HX Maastricht, The Netherlands. [142]Institute of Translational Medicine, University of Liverpool, Liverpool L69 3BX, UK. [143]Max Planck Institute of Psychiatry, 80336 Munich, Germany. [144]Munich Cluster for Systems Neurology (SyNergy), 80336 Munich, Germany. [145]Department of Psychiatry and Psychotherapy, Jena University Hospital, 07743 Jena, Germany. [146]Department of Psychiatry, Queensland Brain Institute and Queensland Centre for Mental Health Research, University of Queensland, Brisbane, Queensland, St Lucia QLD 4072, Australia. [147]Department of Psychiatry and Behavioral Sciences, Johns Hopkins University School of Medicine, Baltimore, Maryland 21205, USA. [148]Department of Psychiatry, Trinity College Dublin, Dublin 2,Ireland. [149]Eli Lilly and Company, Lilly Corporate Center, Indianapolis, 46285 Indiana, USA. [150]Department of Clinical Sciences, Psychiatry, Umeå University, SE-901 87 Umeå, Sweden. [151]DETECT Early Intervention Service for Psychosis, Blackrock, Co. Dublin, Ireland. [152]Centre for Public Health, Institute of Clinical Sciences, Queen's University Belfast, Belfast BT12 6AB, UK. [153]Lawrence Berkeley National Laboratory, University of California at Berkeley, Berkeley, California 94720, USA. [154]Institute of Psychiatry, King's College London, London SE5 8AF, UK. [155]A list of authors and affiliations appear in the Supplementary Information of the original article. [156]Melbourne Neuropsychiatry Centre, University of Melbourne & Melbourne Health, Melbourne, Vic 3053, Australia. [157]Department of Psychiatry, University of Helsinki, P.O. Box 590, FI-00029 HUS, Helsinki, Finland. [158]Public Health Genomics Unit, National Institute for Health and Welfare, P.O. BOX 30, FI-00271 Helsinki, Finland [159]Medical Faculty, University of Belgrade, 11000 Belgrade, Serbia. [160]Department of Psychiatry, University of North Carolina, Chapel Hill, North Carolina 27599-7160, USA. [161]Institute for Molecular Medicine Finland, FIMM, University of Helsinki, P.O. Box 20FI-00014,Helsinki, Finland [162]Department of Epidemiology, Harvard School of Public Health, Boston, Massachusetts 02115, USA. [163]Department of Psychiatry, University of Oxford, Oxford, OX3 7JX, UK. [164]Virginia Institute for Psychiatric and Behavioral Genetics, Virginia Commonwealth University, Richmond, Virginia 23298, USA. [165]Institute for Multiscale Biology, Icahn School of Medicine at Mount Sinai, New York, New York 10029, USA. [166]PharmaTherapeutics Clinical Research, Pfizer Worldwide Research and Development, Cambridge, Massachusetts 02139, USA. [167]Department of Psychiatry and Psychotherapy, University of Gottingen, 37073 Göttingen, Germany. [168]Psychiatry and Psychotherapy Clinic, University of Erlangen, 91054 Erlangen, Germany. [169]Hunter New England Health Service, Newcastle NSW 2308, Australia. [170]School of Biomedical Sciences and Pharmacy, University of Newcastle, Callaghan NSW 2308, Australia. [171]Division of Cancer Epidemiology and Genetics, National Cancer Institute, Bethesda, Maryland 20892, USA. [172]University of Iceland, Landspitali, National University Hospital, 101 Reykjavik, Iceland. [173]Department of Psychiatry and Drug Addiction, Tbilisi State Medical University (TSMU), N33, 0177 Tbilisi, Georgia. [174]Research and Development, Bronx Veterans Affairs Medical Center, New York, New York 10468, USA. [175]Wellcome Trust Centre for Human Genetics, Oxford, OX3 7BN, UK. [176]deCODE

© 2022 Pardiñas AF et al. *JAMA Psychiatry*.

Genetics, 101 Reykjavik, Iceland. [177]Department of Clinical Neurology, Medical University of Vienna, 1090 Wien, Austria. [178]Lieber Institute for Brain Development, Baltimore, Maryland 21205, USA. [179]Department of Medical Genetics, University Medical Centre Utrecht, Universiteitsweg 100, 3584 CG, Utrecht, The Netherlands. [180]Berkshire Healthcare NHS Foundation Trust, Bracknell RG12 1BQ, UK. [181]Section of Psychiatry, University of Verona, 37134 Verona, Italy. [182]Department of Psychiatry, University of Oulu, P.O. BOX 5000, 90014, Finland[183]University Hospital of Oulu, P.O.BOX 20, 90029 OYS, Finland.[184]Molecular and Cellular Therapeutics, Royal College of Surgeons in Ireland, Dublin 2, Ireland. [185]Health Research Board, Dublin 2, Ireland. [186]School of Psychiatry and Clinical Neurosciences, The University of Western Australia, Perth WA6009, Australia. [187]Computational Sciences CoE, Pfizer Worldwide Research and Development, Cambridge, Massachusetts 02139, USA.[188]Human Genetics, Genome Institute of Singapore, A*STAR, Singapore 138672, Singapore. [189]A list of authors and affiliations appearsin the Supplementary Information in https://doi.org/10.1038/nature13595.[190]University College London, London WC1E 6BT, UK. [191]Department of Neuroscience, Icahn School of Medicine at Mount Sinai, New York, New York 10029, USA. [192]Institute of Neuroscience and Medicine (INM-1), Research Center Juelich, 52428 Juelich, Germany. [193]Department of Genetics, The Hebrew University of Jerusalem, 91905 Jerusalem, Israel. [194]Neuroscience Discovery and Translational Area, Pharma Research and Early Development, F. Hoffman-La Roche, CH-4070 Basel, Switzerland. [195]Centre for Clinical Research in Neuropsychiatry, School of Psychiatry and Clinical Neurosciences, The University of Western Australia, Medical Research Foundation Building, Perth WA 6000, Australia. [196]Virginia Institute for Psychiatric and Behavioral Genetics, Departments of Psychiatry and Human and Molecular Genetics, Virginia Commonwealth University, Richmond, Virginia 23298, USA. [197]The Feinstein Institute for Medical Research, Manhasset, New York, 11030 USA. [198]The Hofstra NS-LIJ School of Medicine, Hempstead, New York, 11549 USA. [199]The Zucker Hillside Hospital, Glen Oaks, New York,11004 USA. [200]Saw Swee Hock School of Public Health, National University of Singapore, Singapore 117597, Singapore. [201]Queensland Centre for Mental Health Research, University of Queensland, Brisbane 4076, Queensland, Australia. [202]Center for Human Genetic Research and Department of Psychiatry, Massachusetts General Hospital, Boston, Massachusetts 02114, USA. [203]Department of Child and Adolescent Psychiatry, Erasmus University Medical Centre, Rotterdam 3000, The Netherlands. [204]Department of Complex Trait Genetics, Neuroscience Campus Amsterdam, VU University Medical Center Amsterdam, Amsterdam 1081, The Netherlands. [205]Department of Functional Genomics, Center for Neurogenomics and Cognitive Research, Neuroscience Campus Amsterdam, VU University, Amsterdam 1081, The Netherlands. [206]University of Aberdeen, Institute of Medical Sciences, Aberdeen, AB25 2ZD, UK. [207]Departments of Psychiatry, Neurology, Neuroscience and Institute of Genetic Medicine, Johns Hopkins School of Medicine, Baltimore, Maryland 21205, USA. [208]Department of Clinical Medicine, University of Copenhagen, Copenhagen 2200, Denmark.[209]Departments of Psychiatry and Human Genetics, University of Chicago, Chicago, Illinois 60637, USA

eFigure 1. Mirrored Manhattan plot of the 2 GWAS analysed with the TRS interaction procedure

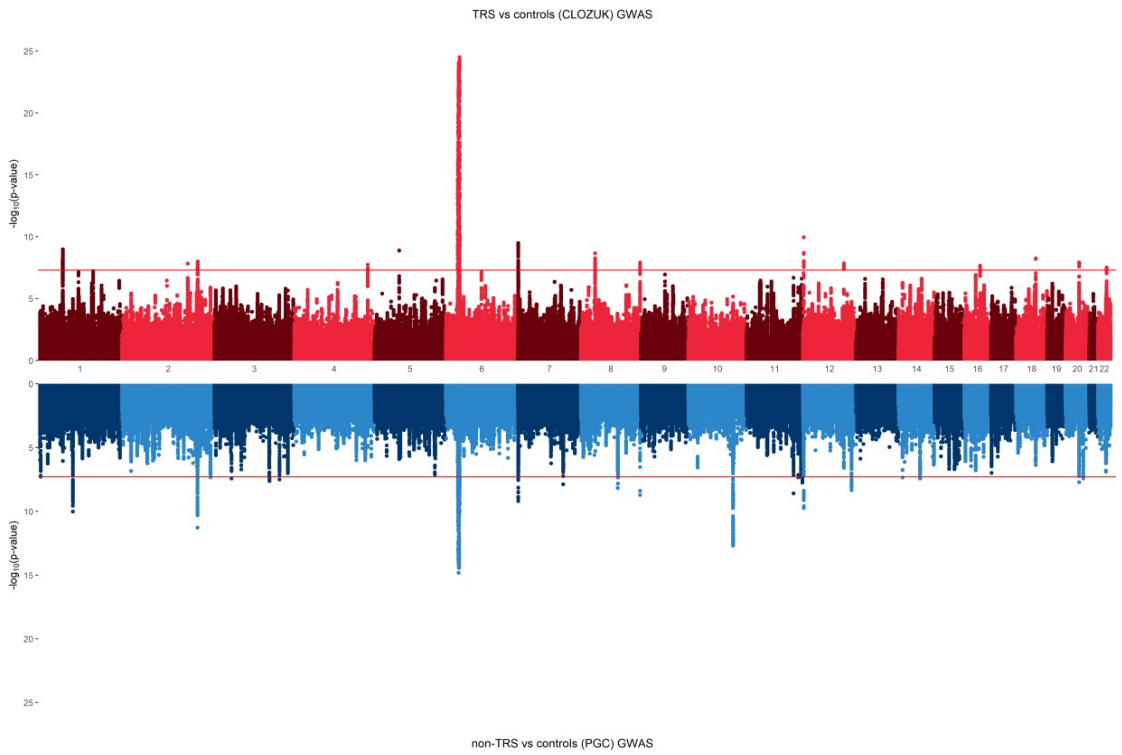

© 2022 Pardiñas AF et al. *JAMA Psychiatry*.

eFigure 2. Q-Q plot of the TRS interaction GWAS

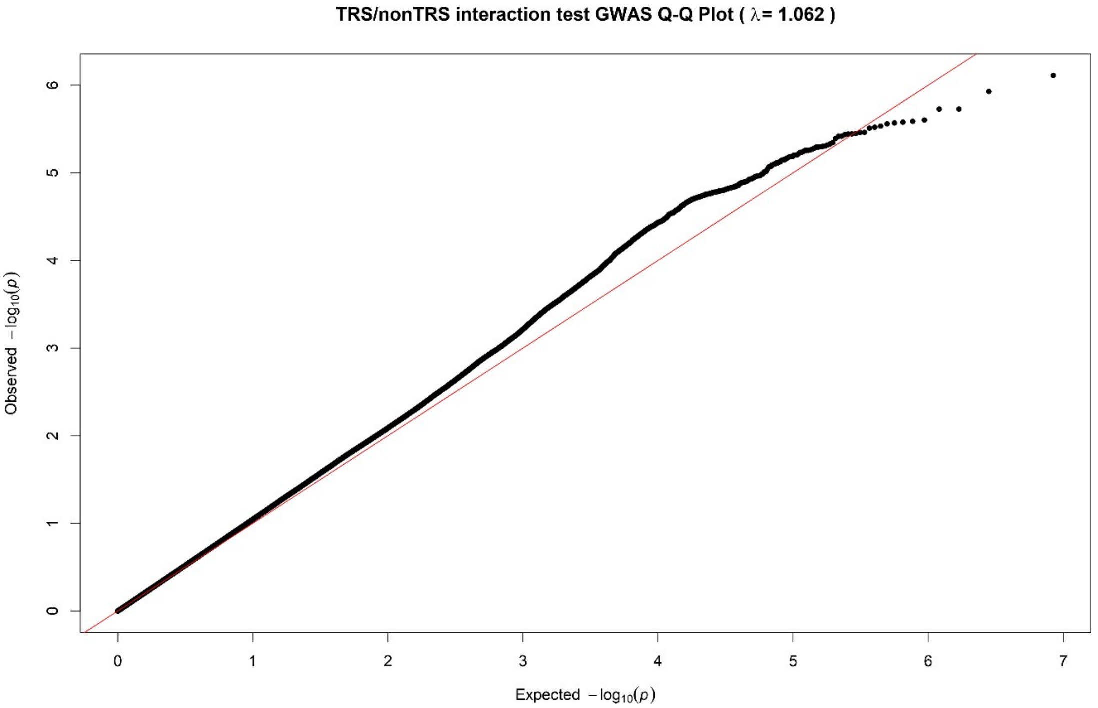

© 2022 Pardiñas AF et al. *JAMA Psychiatry*.

eFigure 3. PRS meta-analysis of CardiffCOGS and STRATA-G cohorts

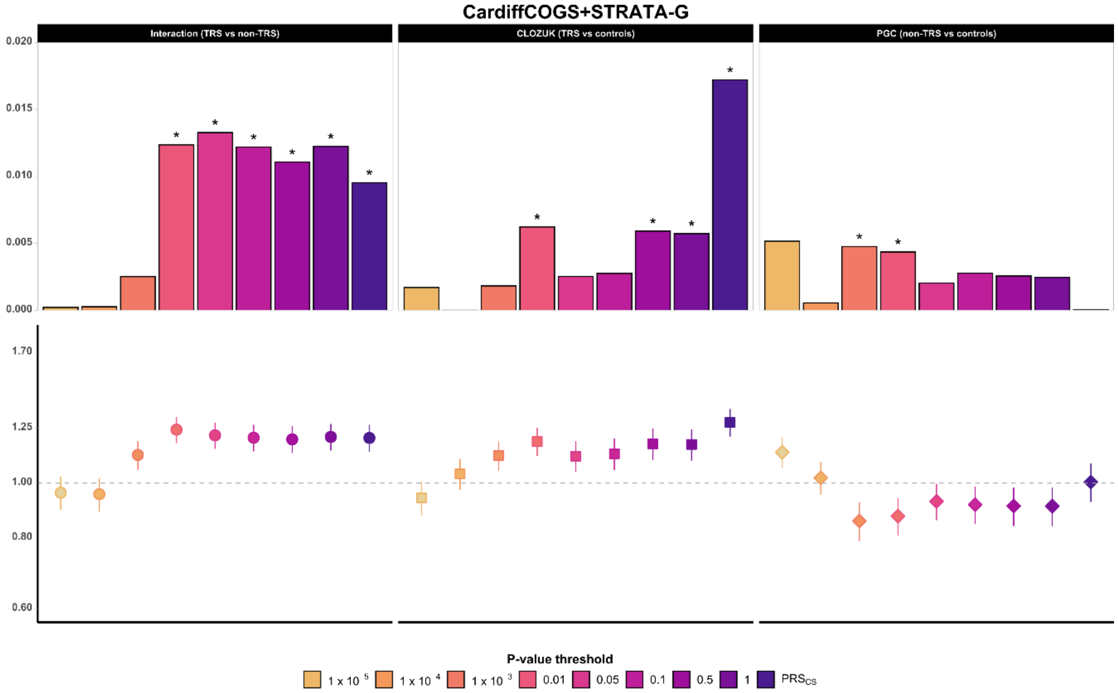

PRS meta-analysis of CardiffCOGS (N_{TRS}=315; N_{NONTRS}=502) and STRATA-G (N_{TRS}=71; N_{NONTRS}=492) cohorts, based on the individual cohort results shown in Figure 2 and detailed in the maintext. Explained variances on the liability scale (upper panel) and effect sizes (lower panel) are shown.Asterisks indicate statistically significant ($p<0.05$) associations between PRS and treatment resistancein schizophrenia, defined as a history of taking clozapine in people with a diagnosis of schizophrenia.

© 2022 Pardiñas AF et al. *JAMA Psychiatry*.

eTable 1. Data sets included in the PGC non-TRS GWAS sample

DATASET ID*	TRS CASES (EXCLUDED)**	NON-TRS CASES	UNAFFECTED CONTROLS
**ABER**	29	691	699
**AJSZ**	*n/a*	896	1595
**ASRB**	33	476	310
**BOCO**	289	1558	2170
**BULS**	*n/a*	527	608
**CATI**	*n/a*	409	392
**CAWS**	*n/a*	476	2936
**CIMS**	*n/a*	71	69
**DENM**	105	387	458
**DUBL**	38	234	860
**EDIN**	*n/a*	368	284
**EGCU**	*n/a*	239	1177
**ERSW**	*n/a*	322	332
**GRAS**	*n/a*	1086	1232
**IRWT**	78	1222	1022
**LACW**	*n/a*	157	466
**LIE2**	*n/a*	137	269
**LIE5**	*n/a*	509	389
**MGS2**	*n/a*	2681	2653
**MSAF**	*n/a*	327	139
**MUNC**	166	271	351
**PEWB**	*n/a*	597	1858
**PEWS**	*n/a*	82	230
**PORT**	22	328	212
**S234**	402	1675	2341
**SWE1**	60	161	214
**SWE5**	433	1368	2617
**SWE6**	228	865	1219
**TOP8**	25	351	402
**UCLA**	*n/a*	705	637
**UCLO**	134	386	494
**UMEB**	*n/a*	375	584
**UMES**	*n/a*	197	713
**ZHH1**	*n/a*	191	190

* For full details see Schizophrenia Working Group of the Psychiatric Genomics Consortium (2014).
** TRS ascertainment could not be carried out in datasets marked with "n/a", as individual-level clinical records were not available.

© 2022 Pardiñas AF et al. *JAMA Psychiatry*.

eTable 2. Data sets included in the STRATA-G sample

DATASET	GEOGRAPHIC AFFILIATION	GENOTYPING CHIP	IMPUTATION BATCH	TRS* CASES	NON-TRS* CASES
**AESOP**	London, UK	Infinium CoreExome-24	2	3	6
**ESS**	Prague, Czech Republic	Infinium Omni 2.5-8	1	1	23
**EUGEI-BOLOGNA & BOFEP**	Bologna, Italy	Human CoreExome-24**	2	0	6
**EUGEI-PARIS**	Paris, France	Human CoreExome-24**	2	6	15
**GAP**	London, UK	Infinium CoreExome-24	2	16	45
**NIFEPS & RGPI**	Belfast, UK	Infinium OmniExpress-24	1	3	8
**PAFIP**	Santander, Spain	Human OmniExpressExome-8	1	21	206
**TIPP**	Lausanne, Switzerland	Infinium OmniExpress-24	1	15	89
**TOP**	Oslo, Norway	Human OmniExpress-12	1	2	72
**UCL**	London, UK	Infinium OmniExpress-24	1	4	22
**TOTAL**				**71**	**492**

* Defined as described in the main text and above, restricted to individuals with schizophrenia at thelast time of follow up.

** Custom chip designed for the EUGEI project (van Os et al., 2014; Mihaljevic et al., 2017).

© 2022 Pardiñas AF et al. *JAMA Psychiatry*.

eTable 3. Polygenic risk score analysis results

Training dataset	*Training phenotype*	*Testing dataset*	*Testing phenotype*	*P-value threshold*	*R2 (%)*	*AUC*	*OR**	*s.e***	*p-value*	*p-value (FDR-corrected)****
TRS Interaction analysis	TRS/non-TRS	CardiffCOGS	Clozapine history	0.00001	0.1853%	0.5201	0.9307	0.0725	0.3218	0.3620
TRS Interaction analysis	TRS/non-TRS	CardiffCOGS	Clozapine history	0.0001	0.1289%	0.5168	0.9418	0.0726	0.4089	0.4089
TRS Interaction analysis	TRS/non-TRS	CardiffCOGS	Clozapine history	0.001	1.0666%	0.5483	1.1881	0.0727	0.0177	0.0439
TRS Interaction analysis	TRS/non-TRS	CardiffCOGS	Clozapine history	0.01	2.0328%	0.5667	1.2741	0.0744	0.0011	0.0099
TRS Interaction analysis	TRS/non-TRS	CardiffCOGS	Clozapine history	0.05	1.0352%	0.5476	1.1862	0.0731	0.0195	0.0439
TRS Interaction analysis	TRS/non-TRS	CardiffCOGS	Clozapine history	0.1	0.7265%	0.5398	1.1538	0.0730	0.0502	0.0645
TRS Interaction analysis	TRS/non-TRS	CardiffCOGS	Clozapine history	0.5	0.7991%	0.5418	1.1607	0.0727	0.0404	0.0606
TRS Interaction analysis	TRS/non-TRS	CardiffCOGS	Clozapine history	1	1.2280%	0.5518	1.2087	0.0728	0.0306	0.0551
TRS Interaction analysis	TRS/non-TRS	CardiffCOGS	Clozapine history	PRS-CS	1.1270%	0.5496	1.2013	0.0746	0.0111	0.0439
CLOZUK	TRS/controls	CardiffCOGS	Clozapine history	0.00001	0.1143%	0.5158	0.9453	0.0725	0.4380	0.4380
CLOZUK	TRS/controls	CardiffCOGS	Clozapine history	0.0001	0.1534%	0.5183	1.0677	0.0726	0.3668	0.4127
CLOZUK	TRS/controls	CardiffCOGS	Clozapine history	0.001	0.7109%	0.5394	1.1534	0.0737	0.0528	0.0950
CLOZUK	TRS/controls	CardiffCOGS	Clozapine history	0.01	1.3627%	0.5546	1.2203	0.0744	0.0075	0.0338
CLOZUK	TRS/controls	CardiffCOGS	Clozapine history	0.05	0.5414%	0.5344	1.1361	0.0754	0.0908	0.1279
CLOZUK	TRS/controls	CardiffCOGS	Clozapine history	0.1	0.5137%	0.5335	1.1344	0.0766	0.0995	0.1279
CLOZUK	TRS/controls	CardiffCOGS	Clozapine history	0.5	0.7470%	0.5404	1.1684	0.0784	0.0471	0.0950
CLOZUK	TRS/controls	CardiffCOGS	Clozapine history	1	0.7172%	0.5396	1.1649	0.0785	0.0516	0.0950
CLOZUK	TRS/controls	CardiffCOGS	Clozapine history	PRS-CS	1.6257%	0.5596	1.2513	0.0768	0.0035	0.0315
PGC	non-TRS/controls	CardiffCOGS	Clozapine history	0.00001	0.4249%	0.5305	1.1171	0.0736	0.1327	0.3981
PGC	non-TRS/controls	CardiffCOGS	Clozapine history	0.0001	0.0006%	0.5011	0.9959	0.0729	0.9553	0.9702
PGC	non-TRS/controls	CardiffCOGS	Clozapine history	0.001	1.2123%	0.5515	0.8319	0.0729	0.0116	0.1044
PGC	non-TRS/controls	CardiffCOGS	Clozapine history	0.01	0.6994%	0.5391	0.8694	0.0729	0.0550	0.2475
PGC	non-TRS/controls	CardiffCOGS	Clozapine history	0.05	0.0724%	0.5126	0.9548	0.0747	0.5362	0.6894
PGC	non-TRS/controls	CardiffCOGS	Clozapine history	0.1	0.1722%	0.5194	0.9299	0.0761	0.3399	0.5098
PGC	non-TRS/controls	CardiffCOGS	Clozapine history	0.5	0.2307%	0.5224	0.9188	0.0769	0.2704	0.4867
PGC	non-TRS/controls	CardiffCOGS	Clozapine history	1	0.2467%	0.5232	0.9160	0.0770	0.2544	0.4867
PGC	non-TRS/controls	CardiffCOGS	Clozapine history	PRS-CS	0.0003%	0.5007	0.9969	0.0824	0.9702	0.9702

© 2022 Pardiñas AF et al. *JAMA Psychiatry*.

Training dataset	*Training phenotype*	*Testing dataset*	*Testing phenotype*	*P-value threshold*	*R2 (%)*	*AUC*	*OR**	*s.e***	*p-value*	*p-value (FDR-corrected)****
TRS Interaction analysis	TRS/non-TRS	STRATA-G	Clozapine history	0.00001	0.0066%	0.5200	1.0574	0.1437	0.6978	0.7850
TRS Interaction analysis	TRS/non-TRS	STRATA-G	Clozapine history	0.0001	0.0003%	0.5022	0.9788	0.1495	0.8862	0.8862
TRS Interaction analysis	TRS/non-TRS	STRATA-G	Clozapine history	0.001	0.0228%	0.5388	0.8706	0.1519	0.3617	0.5426
TRS Interaction analysis	TRS/non-TRS	STRATA-G	Clozapine history	0.01	0.1207%	0.5182	1.0768	0.1443	0.6083	0.7821
TRS Interaction analysis	TRS/non-TRS	STRATA-G	Clozapine history	0.05	0.3508%	0.5461	1.2698	0.1426	0.0940	0.2115
TRS Interaction analysis	TRS/non-TRS	STRATA-G	Clozapine history	0.1	1.0916%	0.5649	1.3482	0.1432	0.0370	0.2073
TRS Interaction analysis	TRS/non-TRS	STRATA-G	Clozapine history	0.5	0.0226%	0.5537	1.3029	0.1455	0.0691	0.2073
TRS Interaction analysis	TRS/non-TRS	STRATA-G	Clozapine history	1	0.0443%	0.5558	1.3270	0.1462	0.0530	0.2073
TRS Interaction analysis	TRS/non-TRS	STRATA-G	Clozapine history	PRS-CS	0.4431%	0.5394	1.1451	0.1412	0.3372	0.5426
CLOZUK	TRS/controls	STRATA-G	Clozapine history	0.00001	0.0920%	0.5310	0.8612	0.1458	0.3055	0.7945
CLOZUK	TRS/controls	STRATA-G	Clozapine history	0.0001	0.1887%	0.5233	0.9015	0.1465	0.4794	0.8629
CLOZUK	TRS/controls	STRATA-G	Clozapine history	0.001	0.0409%	0.5148	0.9280	0.1571	0.6341	0.9069
CLOZUK	TRS/controls	STRATA-G	Clozapine history	0.01	0.0085%	0.5035	1.0131	0.1672	0.9380	0.9484
CLOZUK	TRS/controls	STRATA-G	Clozapine history	0.05	0.0064%	0.5031	1.0120	0.1842	0.9484	0.9484
CLOZUK	TRS/controls	STRATA-G	Clozapine history	0.1	0.0204%	0.5126	1.0809	0.2057	0.7054	0.9069
CLOZUK	TRS/controls	STRATA-G	Clozapine history	0.5	0.4219%	0.5350	1.2505	0.2359	0.3434	0.7945
CLOZUK	TRS/controls	STRATA-G	Clozapine history	1	0.4189%	0.5345	1.2468	0.2376	0.3531	0.7945
CLOZUK	TRS/controls	STRATA-G	Clozapine history	PRS-CS	1.5827%	0.5689	1.4033	0.1975	0.0863	0.7767
PGC	non-TRS/controls	STRATA-G	Clozapine history	0.00001	0.6059%	0.5470	1.2234	0.1746	0.2482	0.7885
PGC	non-TRS/controls	STRATA-G	Clozapine history	0.0001	0.4745%	0.5361	1.1863	0.1772	0.3350	0.7885
PGC	non-TRS/controls	STRATA-G	Clozapine history	0.001	0.0096%	0.5143	1.0313	0.1725	0.8581	0.8581
PGC	non-TRS/controls	STRATA-G	Clozapine history	0.01	0.0607%	0.5020	0.9461	0.1630	0.7342	0.8581
PGC	non-TRS/controls	STRATA-G	Clozapine history	0.05	0.6339%	0.5430	0.8307	0.1751	0.2894	0.7885
PGC	non-TRS/controls	STRATA-G	Clozapine history	0.1	0.1871%	0.5102	0.8752	0.1811	0.4619	0.7885
PGC	non-TRS/controls	STRATA-G	Clozapine history	0.5	0.0882%	0.5143	0.8746	0.1912	0.4833	0.7885
PGC	non-TRS/controls	STRATA-G	Clozapine history	1	0.0714%	0.5122	0.8844	0.1937	0.5257	0.7885
PGC	non-TRS/controls	STRATA-G	Clozapine history	PRS-CS	0.0268%	0.5174	1.0632	0.2437	0.8015	0.8581

© 2022 Pardiñas AF et al. *JAMA Psychiatry*.

Training dataset	*Training phenotype*	*Testing dataset*	*Testing phenotype*	*P-value threshold*	*R2 (%)*	*AUC*	*OR**	*s.e***	*p-value*	*p-value (FDR-corrected)****
TRS Interaction analysis	TRS/non-TRS	Meta-analysis	Clozapine history	0.00001	0.0222%	0.5090	0.9615	0.0645	0.5419	0.5419
TRS Interaction analysis	TRS/non-TRS	Meta-analysis	Clozapine history	0.0001	0.0274%	0.5094	0.9559	0.0650	0.4879	0.5419
TRS Interaction analysis	TRS/non-TRS	Meta-analysis	Clozapine history	0.001	0.2520%	0.5045	1.1209	0.0652	0.0801	0.1030
TRS Interaction analysis	TRS/non-TRS	Meta-analysis	Clozapine history	0.01	1.2357%	0.5593	1.2420	0.0660	0.0010	0.0090
TRS Interaction analysis	TRS/non-TRS	Meta-analysis	Clozapine history	0.05	1.3272%	0.5506	1.2140	0.0649	0.0028	0.0093
TRS Interaction analysis	TRS/non-TRS	Meta-analysis	Clozapine history	0.1	1.2194%	0.5494	1.2022	0.0649	0.0046	0.0093
TRS Interaction analysis	TRS/non-TRS	Meta-analysis	Clozapine history	0.5	1.1068%	0.5468	1.1943	0.0648	0.0062	0.0093
TRS Interaction analysis	TRS/non-TRS	Meta-analysis	Clozapine history	1	1.2238%	0.5495	1.2062	0.0649	0.0039	0.0093
TRS Interaction analysis	TRS/non-TRS	Meta-analysis	Clozapine history	PRS-CS	0.9540%	0.5504	1.2011	0.0657	0.0053	0.0093
CLOZUK	TRS/controls	Meta-analysis	Clozapine history	0.00001	0.1706%	0.5145	0.9417	0.0647	0.3538	0.3980
CLOZUK	TRS/controls	Meta-analysis	Clozapine history	0.0001	0.0007%	0.5084	1.0382	0.0648	0.5632	0.5632
CLOZUK	TRS/controls	Meta-analysis	Clozapine history	0.001	0.1829%	0.5261	1.1177	0.0663	0.0933	0.1439
CLOZUK	TRS/controls	Meta-analysis	Clozapine history	0.01	0.6227%	0.5426	1.1835	0.0676	0.0127	0.0572
CLOZUK	TRS/controls	Meta-analysis	Clozapine history	0.05	0.2539%	0.5269	1.1154	0.0694	0.1156	0.1486
CLOZUK	TRS/controls	Meta-analysis	Clozapine history	0.1	0.2762%	0.5286	1.1263	0.0714	0.0959	0.1439
CLOZUK	TRS/controls	Meta-analysis	Clozapine history	0.5	0.5917%	0.5386	1.1731	0.0741	0.0312	0.0779
CLOZUK	TRS/controls	Meta-analysis	Clozapine history	1	0.5730%	0.5379	1.1699	0.0742	0.0346	0.0779
CLOZUK	TRS/controls	Meta-analysis	Clozapine history	PRS-CS	1.7204%	0.5638	1.2794	0.0715	0.0006	0.0054
PGC	non-TRS/controls	Meta-analysis	Clozapine history	0.00001	0.5163%	0.5350	1.1331	0.0677	0.0648	0.1944
PGC	non-TRS/controls	Meta-analysis	Clozapine history	0.0001	0.0557%	0.5081	1.0214	0.0672	0.7531	0.8472
PGC	non-TRS/controls	Meta-analysis	Clozapine history	0.001	0.4760%	0.5303	0.8570	0.0669	0.0211	0.1899
PGC	non-TRS/controls	Meta-analysis	Clozapine history	0.01	0.4364%	0.5335	0.8742	0.0663	0.0426	0.1917
PGC	non-TRS/controls	Meta-analysis	Clozapine history	0.05	0.2042%	0.5176	0.9282	0.0685	0.2768	0.3559
PGC	non-TRS/controls	Meta-analysis	Clozapine history	0.1	0.2771%	0.5230	0.9159	0.0699	0.2089	0.3134
PGC	non-TRS/controls	Meta-analysis	Clozapine history	0.5	0.2571%	0.5233	0.9110	0.0711	0.1901	0.3134
PGC	non-TRS/controls	Meta-analysis	Clozapine history	1	0.2463%	0.5233	0.9105	0.0713	0.1889	0.3134
PGC	non-TRS/controls	Meta-analysis	Clozapine history	PRS-CS	0.0031%	0.5039	1.0041	0.0779	0.9582	0.9582

* Based on standardised polygenic scores.

** Standard error of the regression beta.

*** Correction performed inside each group formed by training dataset and testing phenotype.

© 2022 Pardiñas AF et al. *JAMA Psychiatry*.

eTable 4. LD-Score and LD-Hub analyses of the TRS GWAS summary statistics

Trait	*PMID*	*Category*	*ethnicity*	*rg*	*se*	*z*	*p*	*pFDR*	*h2_obs*	*h2_obs_se*	*h2_int*	*h2_int_se*
TRS GWAS	this study	-	European	-	-	-	-	-	0.013	0.006	1.036	0.007
Years of schooling 2016	27225129	education	European	-0.660	0.157	-4.202	2.64E-05	0.0007	0.125	0.005	0.946	0.013
College completion	23722424	education	European	-0.691	0.180	-3.832	0.0001	0.0009	0.082	0.006	1.021	0.010
Years of schooling (proxy cognitive performance)	25201988	education	European	-0.639	0.165	-3.875	0.0001	0.0009	0.110	0.008	1.025	0.011
Years of schooling 2013	23722424	education	European	-0.643	0.171	-3.751	0.0002	0.0014	0.086	0.007	1.019	0.010
Intelligence	28530673	cognitive	European	-0.562	0.170	-3.314	0.0009	0.0050	0.191	0.011	1.015	0.011
Former vs Current smoker	20418890	smoking_behaviour	European	-0.658	0.286	-2.298	0.0216	0.1008	0.061	0.013	1.002	0.008
Cigarettes smoked per day	20418890	smoking_behaviour	European	0.661	0.309	2.135	0.0327	0.1308	0.057	0.016	1.007	0.008
Ever vs never smoked	20418890	smoking_behaviour	European	0.413	0.203	2.035	0.0419	0.1467	0.070	0.008	1.007	0.008
Childhood IQ	23358156	education	European	-0.361	0.190	-1.898	0.0577	0.1795	0.273	0.049	1.004	0.010
Bipolar disorder	21926972	psychiatric	European	-0.255	0.158	-1.608	0.1077	0.3016	0.440	0.041	1.022	0.009
Attention deficit hyperactivity disorder	20732625	psychiatric	European	-0.413	0.286	-1.444	0.1487	0.3526	0.256	0.104	1.011	0.008
Attention deficit hyperactivity disorder (GC)	27663945	psychiatric	European	0.493	0.353	1.396	0.1627	0.3526	0.068	0.031	0.996	0.009
Attention deficit hyperactivity disorder (No GC)	27663945	psychiatric	European	0.491	0.353	1.393	0.1637	0.3526	0.069	0.031	1.011	0.009
Depressive symptoms	27089181	psychiatric	European	0.183	0.148	1.230	0.2188	0.4376	0.050	0.004	0.999	0.008
Smoking Initiation	30617275	smoking_behaviour	European	0.546	0.499	1.096	0.2730	0.4801	0.007	0.001	1.077	0.010
Subjective well being	27089181	psychiatric	European	-0.164	0.155	-1.057	0.2905	0.4801	0.026	0.002	0.997	0.008
PGC cross-disorder analysis	23453885	psychiatric	European	-0.146	0.140	-1.040	0.2983	0.4801	0.161	0.014	1.034	0.013
Autism spectrum disorder	30804558	psychiatric	European	-0.177	0.180	-0.985	0.3248	0.4801	0.393	0.055	0.984	0.009
Neuroticism	27089181	personality	European	0.117	0.119	0.983	0.3258	0.4801	0.091	0.007	0.988	0.012
Neo-openness to experience	21173776	personality	European	-0.219	0.255	-0.858	0.3909	0.5473	0.110	0.027	0.991	0.008
Smoking Cessation	30617275	smoking_behaviour	European	-0.704	0.871	-0.808	0.4190	0.5587	0.006	0.003	1.006	0.018
Neo-conscientiousness	21173776	personality	European	0.221	0.308	0.717	0.4735	0.5781	0.071	0.032	1.001	0.008
Neuroticism	24828478	personality	European	0.195	0.273	0.715	0.4749	0.5781	0.013	0.004	1.014	0.008
Schizophrenia	25056061	psychiatric	Mixed	0.059	0.092	0.648	0.5172	0.6034	0.456	0.021	1.064	0.015
Major depressive disorder	22472876	psychiatric	European	-0.109	0.208	-0.522	0.6020	0.6742	0.148	0.029	1.017	0.008
Cigarettes Per Day	30617275	smoking_behaviour	European	-0.159	0.338	-0.470	0.6381	0.6872	0.022	0.013	1.000	0.019
Anorexia Nervosa	24514567	psychiatric	European	-0.048	0.142	-0.340	0.7341	0.7613	0.369	0.032	0.974	0.008
Age of smoking initiation	20418890	smoking_behaviour	European	-0.001	0.290	-0.003	0.9973	0.9973	0.060	0.020	0.999	0.008

© 2022 Pardiñas AF et al. *JAMA Psychiatry*.

eReferences.

1000 Genomes Project Consortium (2015). A global reference for human genetic variation. *Nature;* 526**,** 68-74.

Ajnakina, O., Lally, J., Di Forti, M., Kolliakou, A., Gardner-Sood, P., Lopez-Morinigo, J., Dazzan, P., Pariante, C.M., Mondelli, V., Maccabe, J., David, A.S., Gaughran, F., Murray, R.M., and Vassos, E. (2017). Patterns of illness and care over the 5 years following onset of psychosis in different ethnic groups; the GAP-5 study. *Social Psychiatry and Psychiatric Epidemiology* 52**,** 1101-1111.

Alameda, L., Golay, P., Baumann, P.S., Progin, P., Mebdouhi, N., Elowe, J., Ferrari, C., Do, K.Q., and Conus, P. (2017). Mild Depressive Symptoms Mediate the Impact of Childhood Trauma on Long-TermFunctional Outcome in Early Psychosis Patients. *Schizophr Bull* 43**,** 1027-1035.

Anderson, C.A., Pettersson, F.H., Clarke, G.M., Cardon, L.R., Morris, A.P., and Zondervan, K.T. (2010). Data quality control in genetic case-control association studies. *Nature Protocols* 5**,** 1564–1573.

Andreasen, N.C., Carpenter, W.T., Jr., Kane, J.M., Lasser, R.A., Marder, S.R., and Weinberger, D.R. (2005). Remission in schizophrenia: proposed criteria and rationale for consensus. *Am J Psychiatry* 162**,** 441-449.

Athanasiu, L., Mattingsdal, M., Kahler, A.K., Brown, A., Gustafsson, O., Agartz, I., Giegling, I., Muglia, P., Cichon, S., Rietschel, M., Pietilainen, O.P., Peltonen, L., Bramon, E., Collier, D., Clair, D.S., Sigurdsson, E., Petursson, H., Rujescu, D., Melle, I., Steen, V.M., Djurovic, S., and Andreassen, O.A. (2010). Gene variants associated with schizophrenia in a Norwegian genome-wide study are replicatedin a large European cohort. *J Psychiatr Res* 44**,** 748-753.

Ayesa-Arriola, R., Teran, J.M.P., Moríñigo, J.D.L., Rivero, M.C., Setien-Suero, E., Al-Halabi, S., Cuesta, M.J., David, A.S., and Crespo-Facorro, B. (2018). The dynamic relationship between insight and suicidal behavior in first episode psychosis patients over 3-year follow-up. *Eur Neuropsychopharmacol* 28**,** 1161-1172.

Baumann, P.S., Crespi, S., Marion-Veyron, R., Solida, A., Thonney, J., Favrod, J., Bonsack, C., Do, K.Q., and Conus, P. (2013a). Treatment and Early Intervention in Psychosis Program (TIPP-Lausanne): implementation of an early intervention programme for psychosis in Switzerland. *Early Intervention in Psychiatry* 7**,** 322-328.

Baumann, P.S., Crespi, S., Marion-Veyron, R., Solida, A., Thonney, J., Favrod, J., Bonsack, C., Do, K.Q., and Conus, P. (2013b). Treatment and early intervention in psychosis program (TIPP-Lausanne): Implementation of an early intervention programme for psychosis in Switzerland. *Early Interv Psychiatry* 7**,** 322-328.

Casey, P., and Corvin, A. (2008). The Clinical Impact of Substance Use in Schizophrenia: A Study in an Irish Population. *TSMJ* 9**,** 14-17.

© 2022 Pardiñas AF et al. *JAMA Psychiatry*.

Chang, C.C., Chow, C.C., Tellier, L., Vattikuti, S., Purcell, S.M., and Lee, J.J. (2015). Second-generation PLINK: rising to the challenge of larger and richer datasets. *Gigascience* 4.

Choong, E., Quteineh, L., Cardinaux, J.R., Gholam-Rezaee, M., Vandenberghe, F., Dobrinas, M., Bondolfi, G., Etter, M., Holzer, L., Magistretti, P., Von Gunten, A., Preisig, M., Vollenweider, P., Beckmann, J.S., Pralong, F.P., Waeber, G., Kutalik, Z., Conus, P., Bochud, M., and Eap, C.B. (2013). Influence of CRTC1 polymorphisms on body mass index and fat mass in psychiatric patients and the general adult population. *JAMA Psychiatry* 70**,** 1011-1019.

Choong, E., Solida, A., Lechaire, C., Conus, P., and Eap, C.B. (2008). Follow-up of the metabolic syndrome induced by atypical antipsychotics: recommendations and pharmacogenetics perspectives.*Revue médicale suisse* 4**,** 1994-1996,1998-1999.

Cirulli, E.T., Liu, Q., Zhu, Q., Liu, S., Yao, S., and Han, Y. (2014). Systematic assessment of imputation performance using the 1000 Genomes reference panels. *Briefings in Bioinformatics* 16**,** 549-562.

Conomos, M.P., Miller, M.B., and Thornton, T.A. (2015). Robust inference of population structure for ancestry prediction and correction of stratification in the presence of relatedness. *Genetic epidemiology* 39**,** 276-293.

Conomos, Matthew p., Reiner, Alexander p., Weir, Bruce s., and Thornton, Timothy a. (2016). Model-free Estimation of Recent Genetic Relatedness. *American Journal of Human Genetics* 98**,** 127-148.

Crespo-Facorro, B., Pelayo-Teran, J.M., Perez-Iglesias, R., Ramirez-Bonilla, M., Martinez-Garcia, O., Pardo-Garcia, G., and Vazquez-Barquero, J.L. (2007). Predictors of acute treatment response inpatients with a first episode of non-affective psychosis: sociodemographics, premorbid and clinical variables. *J Psychiatr Res* 41**,** 659-666.

Das, S., Forer, L., Schonherr, S., Sidore, C., Locke, A.E., Kwong, A., Vrieze, S.I., Chew, E.Y., Levy, S., Mcgue, M., Schlessinger, D., Stambolian, D., Loh, P.-R., Iacono, W.G., Swaroop, A., Scott, L.J., Cucca, F., Kronenberg, F., Boehnke, M., Abecasis, G.R., and Fuchsberger, C. (2016). Next-generation genotype imputation service and methods. *Nat Genet* 48**,** 1284-1287.

Dazzan, P., Morgan, K.D., Orr, K., Hutchinson, G., Chitnis, X., Suckling, J., Fearon, P., Mcguire, P.K., Mallett, R.M., Jones, P.B., Leff, J., and Murray, R.M. (2005). Different Effects of Typical and Atypical Antipsychotics on Grey Matter in First Episode Psychosis: the ÆSOP Study. *Neuropsychopharmacology*30**,** 765.

Dean, K., Fearon, P., Morgan, K., Hutchinson, G., Orr, K., Chitnis, X., Suckling, J., Mallet, R., Leff, J., Jones, P.B., Murray, R.M., and Dazzan, P. (2018). Grey matter correlates of minor physical anomalies in the ÆSOP first-episode psychosis study. *British Journal of Psychiatry* 189**,** 221-228.

© 2022 Pardiñas AF et al. *JAMA Psychiatry*.

Debray, T.P.A., Damen, J.a.a.G., Snell, K.I.E., Ensor, J., Hooft, L., Reitsma, J.B., Riley, R.D., and Moons, K.G.M. (2017). A guide to systematic review and meta-analysis of prediction model performance. *BMJ* 356**,** i6460.

Delacretaz, A., Preisig, M., Vandenberghe, F., Saigi Morgui, N., Quteineh, L., Choong, E., Gholam- Rezaee, M., Kutalik, Z., Magistretti, P., Aubry, J.M., Von Gunten, A., Castelao, E., Vollenweider, P., Waeber, G., Conus, P., and Eap, C.B. (2015). Influence of MCHR2 and MCHR2-AS1 Genetic Polymorphisms on Body Mass Index in Psychiatric Patients and In Population-Based Subjects with Present or Past Atypical Depression. *PLoS One* 10**,** e0139155.

Delaneau, O., Zagury, J.-F., and Marchini, J. (2013). Improved whole-chromosome phasing for diseaseand population genetic studies. *Nature Methods* 10**,** 5-6.

Demjaha, A., Lappin, J.M., Stahl, D., Patel, M.X., Maccabe, J.H., Howes, O.D., Heslin, M., Reininghaus, U.A., Donoghue, K., Lomas, B., Charalambides, M., Onyejiaka, A., Fearon, P., Jones, P., Doody, G., Morgan, C., Dazzan, P., and Murray, R.M. (2017). Antipsychotic treatment resistance in first-episode psychosis: prevalence, subtypes and predictors. *Psychological Medicine* 47**,** 1981-1989.

Di Forti, M., Marconi, A., Carra, E., Fraietta, S., Trotta, A., Bonomo, M., Bianconi, F., Gardner-Sood, P.,O'connor, J., Russo, M., Stilo, S.A., Marques, T.R., Mondelli, V., Dazzan, P., Pariante, C., David, A.S., Gaughran, F., Atakan, Z., Iyegbe, C., Powell, J., Morgan, C., Lynskey, M., and Murray, R.M. (2015). Proportion of patients in south London with first-episode psychosis attributable to use of high potencycannabis: a case-control study. *The Lancet Psychiatry* 2**,** 233-238.

Di Forti, M., Morgan, C., Dazzan, P., Pariante, C., Mondelli, V., Marques, T.R., Handley, R., Luzi, S., Russo, M., Paparelli, A., Butt, A., Stilo, S.A., Wiffen, B., Powell, J., and Murray, R.M. (2009). High- potency cannabis and the risk of psychosis. *The British Journal of Psychiatry* 195**,** 488-491.

Faerden, A., Barrett, E.A., Nesvåg, R., Friis, S., Finset, A., Marder, S.R., Ventura, J., Andreassen, O.A., Agartz, I., and Melle, I. (2013). Apathy, poor verbal memory and male gender predict lower psychosocial functioning one year after the first treatment of psychosis. *Psychiatry Research* 210**,** 55-61.

Faerden, A., Nesvag, R., Barrett, E.A., Agartz, I., Finset, A., Friis, S., Rossberg, J.I., and Melle, I. (2008). Assessing apathy: the use of the Apathy Evaluation Scale in first episode psychosis. *Eur Psychiatry* 23**,**33-39.

Fearon, P., Kirkbride, J.B., Morgan, C., Dazzan, P., Morgan, K., Lloyd, T., Hutchinson, G., Tarrant, J., Fung, W.L., Holloway, J., Mallett, R., Harrison, G., Leff, J., Jones, P.B., and Murray, R.M. (2006). Incidence of schizophrenia and other psychoses in ethnic minority groups: results from the MRC AESOP Study. *Psychol Med* 36**,** 1541-1550.

Ge, T., Chen, C.-Y., Ni, Y., Feng, Y.-C.A., and Smoller, J.W. (2019). Polygenic prediction via Bayesian regression and continuous shrinkage priors. *Nature Communications* 10**,** 1776.

© 2022 Pardiñas AF et al. *JAMA Psychiatry*.

Golay, P., Alameda, L., Baumann, P., Elowe, J., Progin, P., Polari, A., and Conus, P. (2016). Duration of untreated psychosis: Impact of the definition of treatment onset on its predictive value over three years of treatment. *J Psychiatr Res* 77**,** 15-21.

Gutierrez-Galve, L., Chu, E.M., Leeson, V.C., Price, G., Barnes, T.R., Joyce, E.M., and Ron, M.A. (2015).A longitudinal study of cortical changes and their cognitive correlates in patients followed up after first-episode psychosis. *Psychol Med* 45**,** 205-216.

Gutierrez-Galve, L., Wheeler-Kingshott, C.A., Altmann, D.R., Price, G., Chu, E.M., Leeson, V.C., Lobo, A., Barker, G.J., Barnes, T.R., Joyce, E.M., and Ron, M.A. (2010). Changes in the frontotemporal cortexand cognitive correlates in first-episode psychosis. *Biol Psychiatry* 68**,** 51-60.

Harel, O. (2009). The estimation of R 2 and adjusted R 2 in incomplete data sets using multiple imputation. *Journal of Applied Statistics* 36**,** 1109-1118.

Howie, B., Fuchsberger, C., Stephens, M., Marchini, J., and Abecasis, G.R. (2012). Fast and accurate genotype imputation in genome-wide association studies through pre-phasing. *Nature Genetics* 44**,** 955-959.

Huddy, V.C., Clark, L., Harrison, I., Ron, M.A., Moutoussis, M., Barnes, T.R., and Joyce, E.M. (2013). Reflection impulsivity and response inhibition in first-episode psychosis: relationship to cannabis use.*Psychol Med* 43**,** 2097-2107.

Huddy, V.C., Hodgson, T.L., Kapasi, M., Mutsatsa, S.H., Harrison, I., Barnes, T.R.E., and Joyce, E.M. (2007). Gaze strategies during planning in first-episode psychosis. *J Abnorm Psychol* 116**,** 589-598.

Jongsma, H.E., Gayer-Anderson, C., Lasalvia, A., Quattrone, D., Mule, A., Szoke, A., Selten, J.P., Turner,C., Arango, C., Tarricone, I., Berardi, D., Tortelli, A., Llorca, P.M., De Haan, L., Bobes, J., Bernardo, M., Sanjuan, J., Santos, J.L., Arrojo, M., Del-Ben, C.M., Menezes, P.R., Velthorst, E., Murray, R.M., Rutten,B.P., Jones, P.B., Van Os, J., Morgan, C., and Kirkbride, J.B. (2018). Treated Incidence of Psychotic Disorders in the Multinational EU-GEI Study. *JAMA Psychiatry* 75**,** 36-46.

Kanahara, N., Yamanaka, H., Suzuki, T., Takase, M., and Iyo, M. (2018). First-episode psychosis in treatment-resistant schizophrenia: a cross-sectional study of a long-term follow-up cohort. *BMC psychiatry* 18**,** 274-274.

Kirkbride, J.B., Fearon, P., Morgan, C., Dazzan, P., Morgan, K., Tarrant, J., Lloyd, T., Holloway, J., Hutchinson, G., Leff, J.P., Mallett, R.M., Harrison, G.L., Murray, R.M., and Jones, P.B. (2006). Heterogeneity in incidence rates of schizophrenia and other psychotic syndromes: findings from the 3-center AeSOP study. *Arch Gen Psychiatry* 63**,** 250-258.

Kolenic, M., Franke, K., Hlinka, J., Matejka, M., Capkova, J., Pausova, Z., Uher, R., Alda, M., Spaniel, F.,and Hajek, T. (2018). Obesity, dyslipidemia and brain

© 2022 Pardiñas AF et al. *JAMA Psychiatry*.

age in first-episode psychosis. *J Psychiatr Res* 99**,**151-158.

Lally, J., Ajnakina, O., Di Forti, M., Trotta, A., Demjaha, A., Kolliakou, A., Mondelli, V., Reis Marques, T.,Pariante, C., Dazzan, P., Shergil, S.S., Howes, O.D., David, A.S., Maccabe, J.H., Gaughran, F., and Murray, R.M. (2016a). Two distinct patterns of treatment resistance: clinical predictors of treatment resistance in first-episode schizophrenia spectrum psychoses. *Psychological Medicine* 46**,** 3231-3240.

Lally, J., Gaughran, F., Timms, P., and Curran, S.R. (2016b). Treatment-resistant schizophrenia: currentinsights on the pharmacogenomics of antipsychotics.

Pharmacogenomics and personalized medicine 9**,** 117.

Lange, E.H., Nesvåg, R., Ringen, P.A., Hartberg, C.B., Haukvik, U.K., Andreassen, O.A., Melle, I., and Agartz, I. (2014). One year follow-up of alcohol and illicit substance use in first-episode psychosis: Does gender matter? *Comprehensive Psychiatry* 55**,** 274-282.

Lee, S.H., Goddard, M.E., Wray, N.R., and Visscher, P.M. (2012). A better coefficient of determinationfor genetic profile analysis. *Genetic epidemiology* 36**,** 214-224.

Legge, S.E., Pardiñas, A.F., Helthuis, M., Jansen, J.A., Jollie, K., Knapper, S., Maccabe, J.H., Rujescu, D.,Collier, D.A., O'donovan, M.C., Owen, M.J., and Walters, J.T.R. (2019). A genome-wide association study in individuals of African ancestry reveals the importance of the Duffy-null genotype in the assessment of clozapine-related neutropenia. *Molecular Psychiatry* 24**,** 328-337.

Lyngstad, S.H., Gardsjord, E.S., Simonsen, C., Engen, M.J., Romm, K.L., Melle, I., and Færden, A. (2018).Consequences of persistent depression and apathy in first-episode psychosis — A one-year follow-upstudy. *Comprehensive Psychiatry* 86**,** 60-66.

Mccarthy, S., Das, S., Kretzschmar, W., Delaneau, O., Wood, A.R., Teumer, A., Kang, H.M., Fuchsberger,C., Danecek, P., Sharp, K., Luo, Y., Sidore, C., Kwong, A., Timpson, N., Koskinen, S., Vrieze, S., Scott, L.J., Zhang, H., Mahajan, A., Veldink, J., Peters, U., Pato, C., Van Duijn, C.M., Gillies, C.E., Gandin, I., Mezzavilla, M., Gilly, A., Cocca, M., Traglia, M., Angius, A., Barrett, J.C., Boomsma, D., Branham, K., Breen, G., Brummett, C.M., Busonero, F., Campbell, H., Chan, A., Chen, S., Chew, E., Collins, F.S., Corbin, L.J., Smith, G.D., Dedoussis, G., Dorr, M., Farmaki, A.-E., Ferrucci, L., Forer, L., Fraser, R.M., Gabriel, S., Levy, S., Groop, L., Harrison, T., Hattersley, A., Holmen, O.L., Hveem, K., Kretzler, M., Lee, J.C., Mcgue, M., Meitinger, T., Melzer, D., Min, J.L., Mohlke, K.L., Vincent, J.B., Nauck, M., Nickerson, D., Palotie, A., Pato, M., Pirastu, N., Mcinnis, M., Richards, J.B., Sala, C., Salomaa, V., Schlessinger, D., Schoenherr, S., Slagboom, P.E., Small, K., Spector, T., Stambolian, D., Tuke, M., Tuomilehto, J., Van Den Berg, L.H., Van Rheenen, W., Volker, U., Wijmenga, C., Toniolo, D., Zeggini, E., Gasparini, P., Sampson,M.G., Wilson, J.F., Frayling, T., De Bakker, P.I.W., Swertz, M.A., Mccarroll, S., Kooperberg, C., Dekker, A., Altshuler, D., Willer, C., Iacono, W., Ripatti, S., et al. (2016). A reference panel of 64,976 haplotypesfor genotype imputation. *Nat Genet* 48**,** 1279-1283.

Mcguffin, P., Farmer, A., and Harvey, I. (1991). A polydiagnostic application of operational criteria in studies of psychotic illness: development and

© 2022 Pardiñas AF et al. *JAMA Psychiatry*.

reliability of the OPCRIT system. *Archives of general psychiatry* 48**,** 764-770.

Melicher, T., Horacek, J., Hlinka, J., Spaniel, F., Tintera, J., Ibrahim, I., Mikolas, P., Novak, T., Mohr, P., and Hoschl, C. (2015). White matter changes in first episode psychosis and their relation to the size ofsample studied: a DTI study. *Schizophr Res* 162**,** 22-28.

Mihaljevic, M., Zeljic, K., Soldatovic, I., Andric, S., Mirjanic, T., Richards, A., Mantripragada, K., Pekmezovic, T., Novakovic, I., and Maric, N.P. (2017). The emerging role of the FKBP5 gene polymorphisms in vulnerability-stress model of schizophrenia: further evidence from a Serbian population. *Eur Arch Psychiatry Clin Neurosci* 267**,** 527-539.

Mikolas, P., Melicher, T., Skoch, A., Matejka, M., Slovakova, A., Bakstein, E., Hajek, T., and Spaniel, F. (2016). Connectivity of the anterior insula differentiates participants with first-episode schizophreniaspectrum disorders from controls: a machine-learning study. *Psychological Medicine* 46**,** 2695-2704.

Morgan, C., Dazzan, P., Morgan, K., Jones, P., Harrison, G., Leff, J., Murray, R., and Fearon, P. (2006). First episode psychosis and ethnicity: initial findings from the AESOP study. *World Psychiatry* 5**,** 40-46.

Morgan, C., Lappin, J., Heslin, M., Donoghue, K., Lomas, B., Reininghaus, U., Onyejiaka, A., Croudace, T., Jones, P.B., Murray, R.M., Fearon, P., Doody, G.A., and Dazzan, P. (2014). Reappraising the long- term course and outcome of psychotic disorders: the AESOP-10 study. *Psychol Med* 44**,** 2713-2726.

Pardiñas, A.F., Holmans, P., Pocklington, A.J., Escott-Price, V., Ripke, S., Carrera, N., Legge, S.E., Bishop,S., Cameron, D., Hamshere, M.L., Han, J., Hubbard, L., Lynham, A., Mantripragada, K., Rees, E., Maccabe, J.H., Mccarroll, S.A., Baune, B.T., Breen, G., Byrne, E.M., Dannlowski, U., Eley, T.C., Hayward,C., Martin, N.G., Mcintosh, A.M., Plomin, R., Porteous, D.J., Wray, N.R., Caballero, A., Geschwind, D.H., Huckins, L.M., Ruderfer, D.M., Santiago, E., Sklar, P., Stahl, E.A., Won, H., Agerbo, E., Als, T.D., Andreassen, O.A., Bækvad-Hansen, M., Mortensen, P.B., Pedersen, C.B., Børglum, A.D., Bybjerg- Grauholm, J., Djurovic, S., Durmishi, N., Pedersen, M.G., Golimbet, V., Grove, J., Hougaard, D.M., Mattheisen, M., Molden, E., Mors, O., Nordentoft, M., Pejovic-Milovancevic, M., Sigurdsson, E., Silagadze, T., Hansen, C.S., Stefansson, K., Stefansson, H., Steinberg, S., Tosato, S., Werge, T., Collier, D.A., Rujescu, D., Kirov, G., Owen, M.J., O'donovan, M.C., and Walters, J.T.R. (2018). Common schizophrenia alleles are enriched in mutation-intolerant genes and in regions under strong background selection. *Nature Genetics* 50**,** 381–389.

Pelayo-Teran, J.M., Perez-Iglesias, R., Ramirez-Bonilla, M., Gonzalez-Blanch, C., Martinez-Garcia, O., Pardo-Garcia, G., Rodriguez-Sanchez, J.M., Roiz-Santianez, R., Tordesillas-Gutierrez, D., Mata, I., Vazquez-Barquero, J.L., and Crespo-Facorro, B. (2008). Epidemiological factors associated with treated incidence of first-episode non-affective psychosis in Cantabria: insights from the Clinical Programme on Early Phases of Psychosis. *Early Interv Psychiatry* 2**,** 178-187.

Peloso, G.M., and Lunetta, K.L. (2011). Choice of population structure informative principal components for adjustment in a case-control study. *BMC*

© 2022 Pardiñas AF et al. *JAMA Psychiatry*.

Genetics 12**,** 64-64.

Peyrot, W.J., and Price, A.L. (2020). Identifying loci with different allele frequencies among cases of eight psychiatric disorders using CC-GWAS. *bioRxiv***,** 2020.2003.2004.977389.

Price, A.L., Weale, M.E., Patterson, N., Myers, S.R., Need, A.C., Shianna, K.V., Ge, D., Rotter, J.I., Torres,E., and Taylor, K.D. (2008). Long-range LD can confound genome scans in admixed populations. *American journal of human genetics* 83**,** 132.

Quteineh, L., Vandenberghe, F., Saigi Morgui, N., Delacretaz, A., Choong, E., Gholam-Rezaee, M., Magistretti, P., Bondolfi, G., Von Gunten, A., Preisig, M., Castelao, E., Vollenweider, P., Waeber, G., Bochud, M., Kutalik, Z., Conus, P., and Eap, C.B. (2015). Impact of HSD11B1 polymorphisms on BMI and components of the metabolic syndrome in patients receiving psychotropic treatments. *Pharmacogenet Genomics* 25**,** 246-258.

Revier, C.J., Reininghaus, U., Dutta, R., Fearon, P., Murray, R.M., Doody, G.A., Croudace, T., Dazzan, P.,Heslin, M., Onyejiaka, A., Kravariti, E., Lappin, J., Lomas, B., Kirkbride, J.B., Donoghue, K., Morgan, C., and Jones, P.B. (2015). Ten-Year Outcomes of First-Episode Psychoses in the MRC ÆSOP-10 Study. *The Journal of Nervous and Mental Disease* 203**,** 379-386.

Schizophrenia Working Group of the Psychiatric Genomics Consortium (2014). Biological insights from108 schizophrenia-associated genetic loci. *Nature* 511**,** 421-427.

Setien-Suero, E., Martinez-Garcia, O., De La Foz, V.O., Vazquez-Bourgon, J., Correa-Ghisays, P., Ferro, A., Crespo-Facorro, B., and Ayesa-Arriola, R. (2018). Age of onset of Cannabis use and cognitive function in first-episode non-affective psychosis patients: Outcome at three-year follow-up. *SchizophrRes* 201**,** 159-166.

Siskind, D., Orr, S., Sinha, S., Yu, O., Brijball, B., Warren, N., Maccabe, J.H., Smart, S.E., and Kisely, S. (2021). Rates of treatment-resistant schizophrenia from first-episode cohorts: systematic review andmeta-analysis. *The British Journal of Psychiatry***,** 1-6.

Spaniel, F., Tintera, J., Rydlo, J., Ibrahim, I., Kasparek, T., Horacek, J., Zaytseva, Y., Matejka, M., Fialova, M., Slovakova, A., Mikolas, P., Melicher, T., Gornerova, N., Hoschl, C., and Hajek, T. (2016). Altered Neural Correlate of the Self-Agency Experience in First-Episode Schizophrenia-Spectrum Patients: An fMRI Study. *Schizophr Bull* 42**,** 916-925.

Tarricone, I., Mimmi, S., Paparelli, A., Rossi, E., Mori, E., Panigada, S., Carchia, G., Bandieri, V., Michetti, R., Minenna, G., Boydell, J., Morgan, C., and Berardi, D. (2012). First-episode psychosis at the West Bologna Community Mental Health Centre: results of an 8-year prospective study. *Psychol Med* 42**,** 2255-2264.

© 2022 Pardiñas AF et al. *JAMA Psychiatry*.

Turkington, A., Mulholland, C.C., Rushe, T.M., Anderson, R., Mccaul, R., Barrett, S.L., Barr, R.S., and Cooper, S.J. (2018). Impact of persistent substance misuse on 1-year outcome in first-episode psychosis. *British Journal of Psychiatry* 195**,** 242-248.

Üçok, A., Çıkrıkçılı, U., Ergül, C., Tabak, Ö., Salaj, A., Karabulut, S., and Correll, C.U. (2016). Correlates of Clozapine Use after a First Episode of Schizophrenia: Results From a Long-term Prospective Study. *CNS Drugs* 30**,** 997-1006.

Üçok, A., Polat, A., Cakir, S., and Genc, A. (2006). One year outcome in first episode schizophrenia. Predictors of relapse. *Eur Arch Psychiatry Clin Neurosci* 256**,** 37-43.

Üçok, A., Polat, A., Genc, A., Cakir, S., and Turan, N. (2004). Duration of untreated psychosis may predict acute treatment response in first-episode schizophrenia. *J Psychiatr Res* 38**,** 163-168.

Üçok, A., Serbest, S., and Kandemir, P.E. (2011). Remission after first-episode schizophrenia: results ofa long-term follow-up. *Psychiatry Res* 189**,** 33-37.

Van Os, J., Rutten, B.P., Myin-Germeys, I., Delespaul, P., Viechtbauer, W., Van Zelst, C., Bruggeman, R., Reininghaus, U., Morgan, C., Murray, R.M., Di Forti, M., Mcguire, P., Valmaggia, L.R., Kempton, M.J.,Gayer-Anderson, C., Hubbard, K., Beards, S., Stilo, S.A., Onyejiaka, A., Bourque, F., Modinos, G., Tognin, S., Calem, M., O'donovan, M.C., Owen, M.J., Holmans, P., Williams, N., Craddock, N., Richards, A., Humphreys, I., Meyer-Lindenberg, A., Leweke, F.M., Tost, H., Akdeniz, C., Rohleder, C., Bumb, J.M., Schwarz, E., Alptekin, K., Üçok, A., Saka, M.C., Atbaşoğlu, E.C., Gülöksüz, S., Gumus-Akay, G., Cihan, B.,Karadağ, H., Soygür, H., Cankurtaran, E., Ulusoy, S., Akdede, B., Binbay, T., Ayer, A., Noyan, H., Karadayı, G., Akturan, E., Ulaş, H., Arango, C., Parellada, M., Bernardo, M., Sanjuán, J., Bobes, J., Arrojo,M., Santos, J.L., Cuadrado, P., Rodríguez Solano, J.J., Carracedo, A., García Bernardo, E., Roldán, L., López, G., Cabrera, B., Cruz, S., Díaz Mesa, E.M., Pouso, M., Jiménez, E., Sánchez, T., Rapado, M., González, E., Martínez, C., Sánchez, E., Olmeda, M.S., De Haan, L., Velthorst, E., Van Der Gaag, M., Selten, J.P., Van Dam, D., Van Der Ven, E., Van Der Meer, F., Messchaert, E., Kraan, T., Burger, N., Leboyer, M., Szoke, A., Schürhoff, F., Llorca, P.M., Jamain, S., Tortelli, A., Frijda, F., Vilain, J., Galliot, A.M., Baudin, G., Ferchiou, A., et al. (2014). Identifying gene-environment interactions in schizophrenia: contemporary challenges for integrated, large-scale investigations. *Schizophr Bull* 40**,** 729-736.

Vandenberghe, F., Gholam-Rezaee, M., Saigi-Morgui, N., Delacretaz, A., Choong, E., Solida-Tozzi, A., Kolly, S., Thonney, J., Gallo, S.F., Hedjal, A., Ambresin, A.E., Von Gunten, A., Conus, P., and Eap, C.B. (2015). Importance of early weight changes to predict long-term weight gain during psychotropic drug treatment. *J Clin Psychiatry* 76**,** e1417-1423.

Viechtbauer, W. (2010). Conducting meta-analyses in R with the metafor package. *Journal of StatisticalSoftware* 36**,** 1-48.

Willer, C.J., Li, Y., and Abecasis, G.R. (2010). METAL: fast and efficient meta-analysis of genomewide association scans. *Bioinformatics* 26**,** 2190-2191.

© 2022 Pardiñas AF et al. *JAMA Psychiatry*.

Zimbron, J., Stahl, D., Hutchinson, G., Dazzan, P., Morgan, K., Doody, G.A., Jones, P.B., Murray, R.M., Fearon, P., Morgan, C., and Maccabe, J.H. (2014). Pre-morbid fertility in psychosis: Findings from the AESOP first episode study. *Schizophrenia Research* 156**,** 168-173

© 2022 Pardiñas AF et al. *JAMA Psychiatry*.
