# Supplementary material for: Interaction Testing and Polygenic Risk Scoring to Estimate the Association of Common Genetic Variants With Treatment Resistance in Schizophrenia
Source: JAMA Psychiatry. 2022 Jan 12;79(3):1–11. doi: 10.1001/jamapsychiatry.2021.3799 (PMC8756361; doi:10.1001/jamapsychiatry.2021.3799)
Supplement: Supplement 2. — Nonauthor collaborators. Collaborators of the Genetics Workstream of the Schizophrenia Treatment Resistance and Therapeutic Advances (STRATA) Consortium and the Schizophrenia Working Group of the Psychiatric Genomics Consortium (PGC). [file jamapsychiatry-e213799-s002.pdf]

Supplemental Online Content: Nonauthor Collaborators

*Indicates required information. Only first name, last name, and suffix will appear in PubMed.

*Group Name(s): Schizophrenia Working Group of the Psychiatric Genomics Consortium (PGC) and the Genetics Workstream of the Schizophrenia Treatment Resistance and Therapeutic Advances (STRATA) Consortium							
***First Name and Middle Initial(s)**	***Last Name**	***Suffix (eg, Jr, III)**	Academic Degrees	Institution	Location (city, state/province, country)	Role or Contribution, eg, chair, principal investigator	Group (if more than 1 Group listed in the byline) and/or Subgroup (eg, Steering Committee)
Stephan	Ripke						PGC
Benjamin M.	Neale						PGC
Kai-How	Farh						PGC
Phil	Lee						PGC
Brendan	Bulik-Sullivan						PGC
David A.	Collier						PGC
Hailiang	Huang						PGC
Tune H.	Pers						PGC
Ingrid	Agartz						PGC
Esben	Agerbo						PGC
Margot	Albus						PGC
Madeline	Alexander						PGC
Farooq	Amin						PGC
Silviu A.	Bacanu						PGC
Martin	Begemann						PGC
Richard A .	Belliveau	Jr					PGC
Judit	Bene						PGC
Sarah E.	Bergen						PGC
Elizabeth	Bevilacqua						PGC
Donald W.	Black						PGC
Richard	Bruggeman						PGC
Nancy G.	Buccola						PGC
Randy L.	Buckner						PGC
William	Byerley						PGC
Wiepke	Cahn						PGC
Guiqing	Cai						PGC
Dominique	Campion						PGC
Rita M.	Cantor						PGC
Vaughan J.	Carr						PGC
Noa	Carrera						PGC
Stanley V.	Catts						PGC
Kimberly D.	Chambert						PGC

Supplemental Online Content: Nonauthor Collaborators

*Indicates required information. Only first name, last name, and suffix will appear in PubMed.

*First Name and Middle Initial(s)	*Last Name	*Suffix (eg, Jr, III)	Academic Degrees	Institution	Location (city, state/province, country)	Role or Contribution, eg, chair, principal investigator	Group (if more than 1 Group listed in the byline) and/or Subgroup (eg, Steering Committee)
Raymond C. K.	Chan						PGC
Ronald Y. L.	Chen						PGC
Eric Y. H.	Chen						PGC
Wei	Cheng						PGC
Eric F. C.	Cheung						PGC
Siow Ann	Chong						PGC
C. Robert	Cloninger						PGC
David	Cohen						PGC
Nadine	Cohen						PGC
Paul	Cormican						PGC
Nick	Craddock						PGC
James J.	Crowley						PGC
David	Curtis						PGC
Michael	Davidson						PGC
Kenneth L.	Davis						PGC
Franziska	Degenhardt						PGC
Jurgen Del	Favero						PGC
Lynn E.	DeLisi						PGC
Ditte	Demontis						PGC
Dimitris	Dikeos						PGC
Timothy	Dinan						PGC
Srdjan	Djurovic						PGC
Gary	Donohoe						PGC
Elodie	Drapeau						PGC
Jubao	Duan						PGC
Frank	Dudbridge						PGC
Naser	Durmishi						PGC
Peter	Eichhammer						PGC
Johan	Eriksson						PGC
Valentina	Escott-Price						PGC
Laurent	Essioux						PGC
Martilias S.	Farrell						PGC
Lude	Franke						PGC
Robert	Freedman						PGC

Supplemental Online Content: Nonauthor Collaborators

*Indicates required information. Only first name, last name, and suffix will appear in PubMed.

*First Name and Middle Initial(s)	*Last Name	*Suffix (eg, Jr, III)	Academic Degrees	Institution	Location (city, state/province, country)	Role or Contribution, eg, chair, principal investigator	Group (if more than 1 Group listed in the byline) and/or Subgroup (eg, Steering Committee)
Nelson B.	Freimer						PGC
Marion	Friedl						PGC
Joseph I.	Friedman						PGC
Menachem	Fromer						PGC
Giulio	Genovese						PGC
Lyudmila	Georgieva						PGC
Elliot S.	Gershon						PGC
Ina	Giegling						PGC
Paola	Giusti-Rodríguez						PGC
Stephanie	Godard						PGC
Jacqueline I.	Goldstein						PGC
Vera	Golimbet						PGC
Srihari	Gopal						PGC
Jacob	Gratten						PGC
Lieuwe de	Haan						PGC
Christian	Hammer						PGC
Marian L.	Hamshere						PGC
Mark	Hansen						PGC
Thomas	Hansen						PGC
Vahram	Haroutunian						PGC
Annette M.	Hartmann						PGC
Frans A.	Henskens						PGC
Stefan	Herms						PGC
Joel N.	Hirschhorn						PGC
Per	Hoffmann						PGC
Andrea	Hofman						PGC
Mads V.	Hollegaard						PGC
David M.	Hougaard						PGC
Masashi	Ikeda						PGC
Inge	Joa						PGC
Antonio	Julià						PGC
René S.	Kahn						PGC
Luba	Kalaydjieva						PGC

*Indicates required information. Only first name, last name, and suffix will appear in PubMed.

*First Name and Middle Initial(s)	*Last Name	*Suffix (eg, Jr, III)	Academic Degrees	Institution	Location (city, state/province, country)	Role or Contribution, eg, chair, principal investigator	Group (if more than 1 Group listed in the byline) and/or Subgroup (eg, Steering Committee)
Sena	Karachanak-Yankova						PGC
Juha	Karjalainen						PGC
David	Kavanagh						PGC
Matthew C.	Keller						PGC
James L.	Kennedy						PGC
Andrey	Khrunin						PGC
Yunjung	Kim						PGC
Janis	Klovins						PGC
James A.	Knowles						PGC
Bettina	Konte						PGC
Vaidutis	Kucinskas						PGC
Zita Ausrele	Kucinskiene						PGC
Hana	Kuzelova-Ptackova						PGC
Anna K.	Kähler						PGC
Claudine	Laurent						PGC
Jimmy Lee Chee	Keong						PGC
S. Hong	Lee						PGC
Bernard	Lerer						PGC
Miaoxin	Li						PGC
Tao	Li						PGC
Kung-Yee	Liang						PGC
Jeffrey	Lieberman						PGC
Svetlana	Limborska						PGC
Carmel M.	Loughland						PGC
Jan	Lubinski						PGC
Jouko	Lönnqvist						PGC
Milan	Macek	Jr					PGC
Patrik K. E.	Magnusson						PGC
Brion S.	Maher						PGC
Wolfgang	Maier						PGC
Jacques	Mallet						PGC
Sara	Marsal						PGC

Supplemental Online Content: Nonauthor Collaborators

*Indicates required information. Only first name, last name, and suffix will appear in PubMed.

*First Name and Middle Initial(s)	*Last Name	*Suffix (eg, Jr, III)	Academic Degrees	Institution	Location (city, state/province, country)	Role or Contribution, eg, chair, principal investigator	Group (if more than 1 Group listed in the byline) and/or Subgroup (eg, Steering Committee)
Manuel	Mattheisen						PGC
Morten	Mattingsdal						PGC
Robert W.	McCarley						PGC
Colm	McDonald						PGC
Andrew M.	McIntosh						PGC
Sandra	Meier						PGC
Carin J.	Meijer						PGC
Bela	Melegh						PGC
Ingrid	Melle						PGC
Raquelle I.	Mesholam-Gately						PGC
Andres	Metspalu						PGC
Patricia T.	Michie						PGC
Lili	Milani						PGC
Vihra	Milanova						PGC
Younes	Mokrab						PGC
Derek W.	Morris						PGC
Ole	Mors						PGC
Kieran C.	Murphy						PGC
Inez	Myin-Germeys						PGC
Bertram	Müller-Myhsok						PGC
Mari	Nelis						PGC
Igor	Nenadic						PGC
Deborah A.	Nertney						PGC
Gerald	Nestadt						PGC
Kristin K.	Nicodemus						PGC
Liene	Nikitina-Zake						PGC
Laura	Nisenbaum						PGC
Annelie	Nordin						PGC
Eadbhard	O'Callaghan						PGC
Colm	O'Dushlaine						PGC
F. Anthony	O'Neill						PGC
Sang-Yun	Oh						PGC
Ann	Olincy						PGC

Supplemental Online Content: Nonauthor Collaborators

*Indicates required information. Only first name, last name, and suffix will appear in PubMed.

*First Name and Middle Initial(s)	*Last Name	*Suffix (eg, Jr, III)	Academic Degrees	Institution	Location (city, state/province, country)	Role or Contribution, eg, chair, principal investigator	Group (if more than 1 Group listed in the byline) and/or Subgroup (eg, Steering Committee)
Line	Olsen						PGC
Jim Van	Os						PGC
Christos	Pantelis						PGC
George N.	Papadimitriou						PGC
Sergi	Papiol						PGC
Elena	Parkhomenko						PGC
Michele T.	Pato						PGC
Tiina	Paunio						PGC
Milica	Pejovic-Milovancevic						PGC
Diana O.	Perkins						PGC
Olli	Pietiläinen						PGC
Jonathan	Pimm						PGC
Andrew J.	Pocklington						PGC
John	Powell						PGC
Alkes	Price						PGC
Ann E.	Pulver						PGC
Shaun M.	Purcell						PGC
Digby	Quested						PGC
Henrik B.	Rasmussen						PGC
Abraham	Reichenberg						PGC
Mark A.	Reimers						PGC
Alexander L.	Richards						PGC
Joshua L.	Roffman						PGC
Panos	Roussos						PGC
Douglas M.	Ruderfer						PGC
Veikko	Salomaa						PGC
Alan R.	Sanders						PGC
Ulrich	Schall						PGC
Christian R.	Schubert						PGC
Thomas G.	Schulze						PGC
Sibylle G.	Schwab						PGC
Edward M.	Scolnick						PGC
Rodney J.	Scott						PGC

Supplemental Online Content: Nonauthor Collaborators

*Indicates required information. Only first name, last name, and suffix will appear in PubMed.

*First Name and Middle Initial(s)	*Last Name	*Suffix (eg, Jr, III)	Academic Degrees	Institution	Location (city, state/province, country)	Role or Contribution, eg, chair, principal investigator	Group (if more than 1 Group listed in the byline) and/or Subgroup (eg, Steering Committee)
Larry J.	Seidman						PGC
Jianxin	Shi						PGC
Engilbert	Sigurdsson						PGC
Teimuraz	Silagadze						PGC
Jeremy M.	Silverman						PGC
Kang	Sim						PGC
Petr	Slominsky						PGC
Jordan W.	Smoller						PGC
Hon-Cheong	So						PGC
Chris C. A.	Spencer						PGC
Eli A.	Stahl						PGC
Hreinn	Stefansson						PGC
Stacy	Steinberg						PGC
Elisabeth	Stogmann						PGC
Richard E.	Straub						PGC
Eric	Strengman						PGC
Jana	Strohmaier						PGC
T. Scott	Stroup						PGC
Mythily	Subramaniam						PGC
Jaana	Suvisaari						PGC
Dragan M.	Svrakic						PGC
Jin P.	Szatkiewicz						PGC
Erik	Söderman						PGC
Srinivas	Thirumalai						PGC
Draga	Toncheva						PGC
Sarah	Tosato						PGC
Juha	Veijola						PGC
John	Waddington						PGC
Dermot	Walsh						PGC
Dai	Wang						PGC
Qiang	Wang						PGC
Bradley T.	Webb						PGC
Mark	Weiser						PGC
Dieter B.	Wildenauer						PGC

Supplemental Online Content: Nonauthor Collaborators

*Indicates required information. Only first name, last name, and suffix will appear in PubMed.

*First Name and Middle Initial(s)	*Last Name	*Suffix (eg, Jr, III)	Academic Degrees	Institution	Location (city, state/province, country)	Role or Contribution, eg, chair, principal investigator	Group (if more than 1 Group listed in the byline) and/or Subgroup (eg, Steering Committee)
Nigel M.	Williams						PGC
Stephanie	Williams						PGC
Stephanie H.	Witt						PGC
Aaron R.	Wolen						PGC
Emily H. M.	Wong						PGC
Brandon K.	Wormley						PGC
Hualin Simon	Xi						PGC
Clement C.	Zai						PGC
Xuebin	Zheng						PGC
Fritz	Zimprich						PGC
Naomi R.	Wray						PGC
Kari	Stefansson						PGC
Peter M.	Visscher						PGC
Rolf	Adolfsson						PGC
Douglas H. R.	Blackwood						PGC
Elvira	Bramon						PGC
Joseph D.	Buxbaum						PGC
Anders D.	Børglum						PGC
Sven	Cichon						PGC
Ariel	Darvasi						PGC
Enrico	Domenici						PGC
Hannelore	Ehrenreich						PGC
Tõnu	Esko						PGC
Pablo V.	Gejman						PGC
Michael	Gill						PGC
Hugh	Gurling						PGC
Christina M.	Hultman						PGC
Nakao	Iwata						PGC
Assen V.	Jablensky						PGC
Erik G.	Jönsson						PGC
Kenneth S.	Kendler						PGC
George	Kirov						PGC
Jo	Knight						PGC
Todd	Lencz						PGC

Supplemental Online Content: Nonauthor Collaborators

*Indicates required information. Only first name, last name, and suffix will appear in PubMed.

*First Name and Middle Initial(s)	*Last Name	*Suffix (eg, Jr, III)	Academic Degrees	Institution	Location (city, state/province, country)	Role or Contribution, eg, chair, principal investigator	Group (if more than 1 Group listed in the byline) and/or Subgroup (eg, Steering Committee)
Douglas F.	Levinson						PGC
Qingqin S.	Li						PGC
Jianjun	Liu						PGC
Anil K.	Malhotra						PGC
Steven A.	McCarroll						PGC
Jennifer L.	Moran						PGC
Preben B.	Mortensen						PGC
Markus M.	Nöthen						PGC
Roel A.	Ophoff						PGC
Aarno	Palotie						PGC
Tracey L.	Petryshen						PGC
Danielle	Posthuma						PGC
Brien P.	Riley						PGC
Pak C.	Sham						PGC
Pamela	Sklar						PGC
David St	Clair						PGC
Daniel R.	Weinberger						PGC
Jens R.	Wendland						PGC
Thomas	Werge						PGC
Mark J.	Daly						PGC
Deborah	Agbedjro						STRATA
Daniel	Stahl						STRATA
Shitij	Kapur						STRATA
Edward	Millgate						STRATA
Adrianna	Kepinska						STRATA
Eugenia	Kravariti						STRATA
